# Supplementary material for: Precision therapeutic targeting of human cancer cell motility
Source: Nat Commun. 2018 Jun 22;9:2454. doi: 10.1038/s41467-018-04465-5 (PMC6014988; doi:10.1038/s41467-018-04465-5)
Supplement: Supplementary file 1 — Supplementary Information [file 41467_2018_4465_MOESM1_ESM.pdf]

Supplementary Information

Precision Therapeutic Targeting of Human Cancer Cell Motility

Xu et al.

Supplementary Figure 1a.

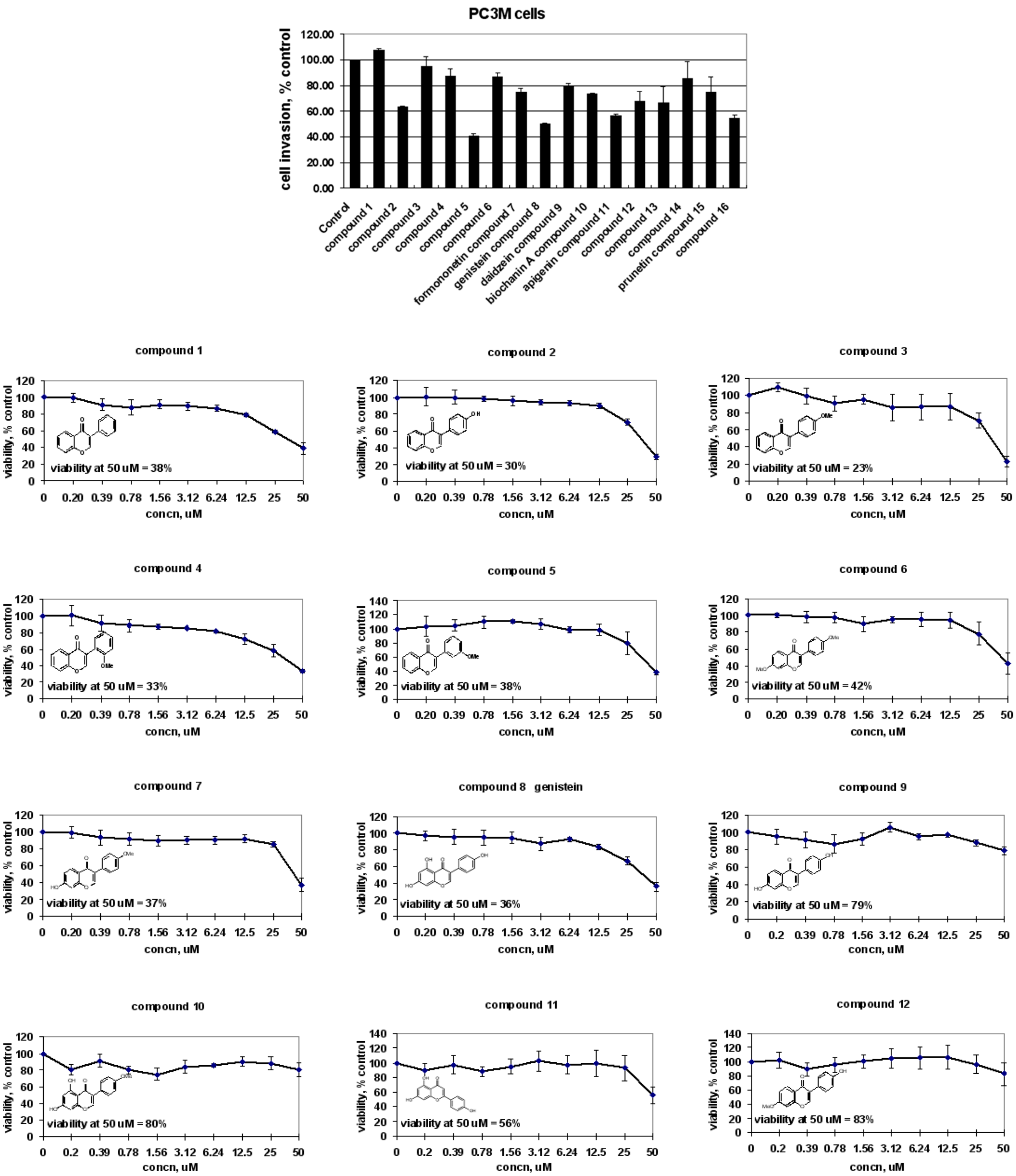

compound 13

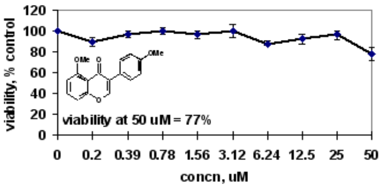

compound 14

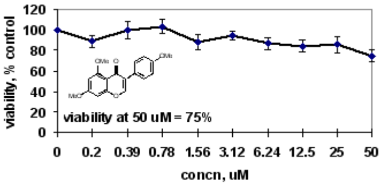

compound 15

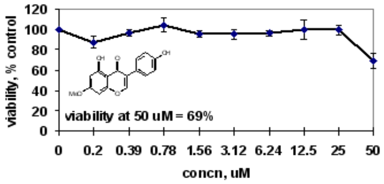

compound 16

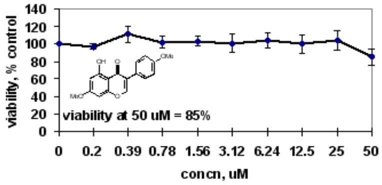

Supplementary Figure 1b.

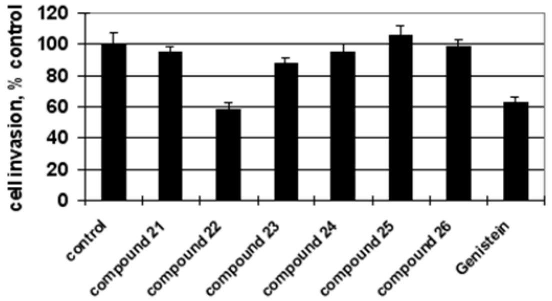

compound 21

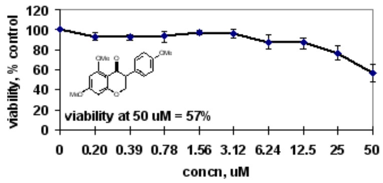

compound 22

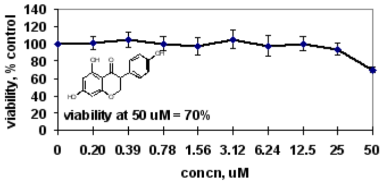

compound 23

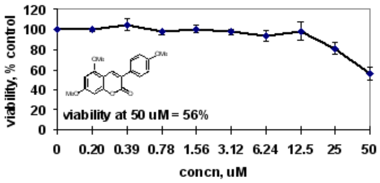

compound 24

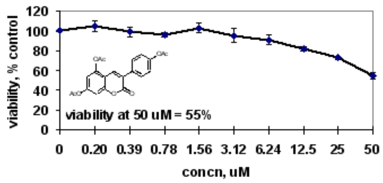

compound 25

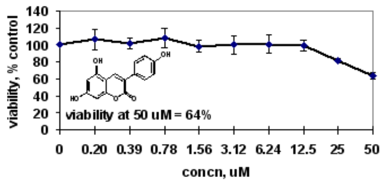

compound 26

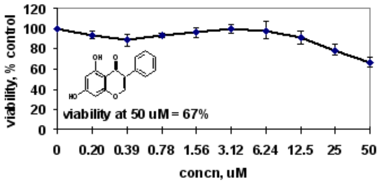

Supplementary Figure 1c.

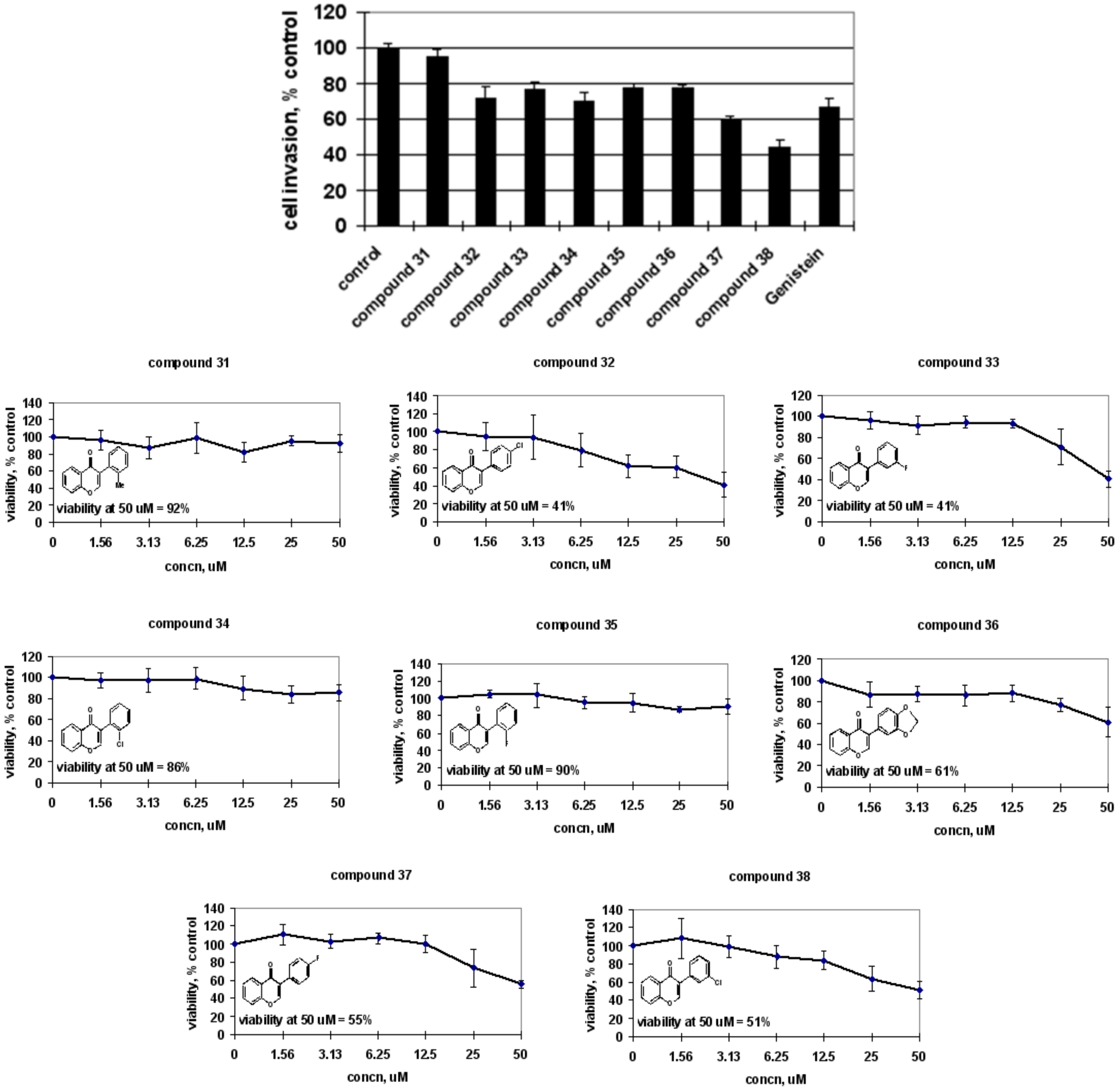

Supplementary Figure 1d.

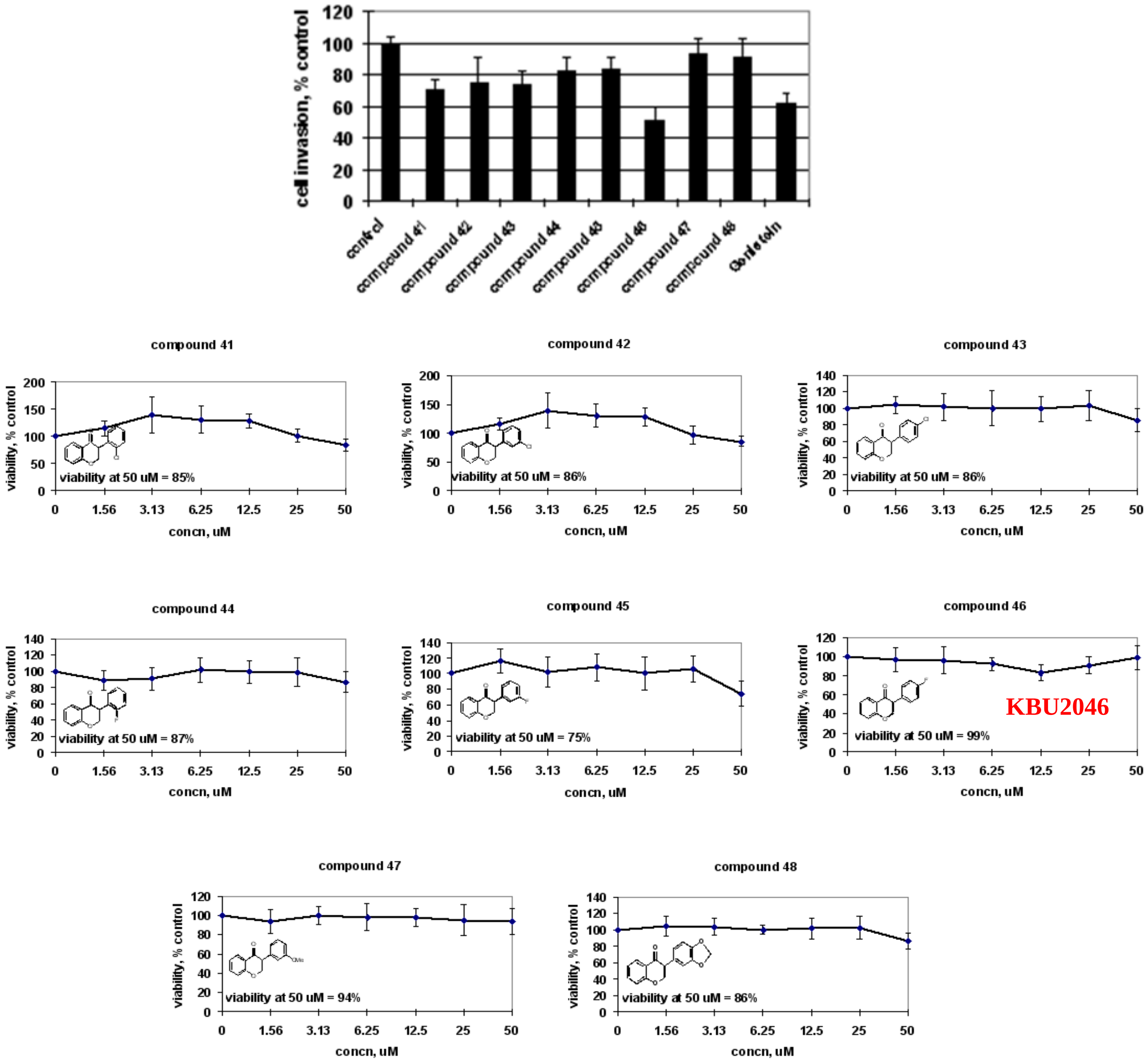

Supplementary Figure 1. Synthesis of KBU2046. Following the synthetic strategy outlined in manuscript Fig. 1 and Supplementary Note 1, we used 4´,5,7-trihydroxyisoflavone as our chemical scaffold.

Supplementary Figure 1a. Synthetic round #1. As this scaffold had anti-invasion efficacy,

we first evaluated which of its chemical fragments were necessary for activity by synthesizing a set of compounds lacking individual functional groups, and assessing their effects upon cell invasion and cell growth inhibition. Key informative findings include: the ring C4´-hydroxyl group is necessary for activity (compare compounds 1 and 2) and removal of the C7-hydroxyl group (which mediates binding to the ER) does not affect activity (compare compounds 2 and 8). Other relevant findings: movement of the C4´-hydroxyl is associated with retention of activity (compare compounds 2 and 5), and it is possible to achieve growth inhibition while having minimal impact upon invasion; consider compounds 1, 3, 4, and 6. Note, demethylation within the cell cannot be predicted. Therefore, we only considered loss of function to be informative for methylated compounds. For example, consider compound 7, where methylation of the C4´-hydroxyl group leads to loss of invasion (compare to compound 8). This adds further evidence of the importance of the C4´-hydroxyl for activity. In contrast, while the C7- and C4´-hydroxyl groups of compound 16 are methylated, it retains anti-invasion activity, indicating that demethylation within the cell could possibly influence the results. For cell invasion, PC3-M cells were treated with 10 μM compound for a total of 3 days, and cell invasion assays were run at the end of the 3 day period, in the presence of compound. Values are the mean±SEM of three separate experiments, each run in replicates of N=3. Three day MTT cell growth inhibition assays were performed with PC3-M cells. Values are the mean±SEM of a single experiment, in replicates of N=4, repeated at least once (also N=4).

Supplementary Figure 1b. Synthetic round #2. Our initial structure-activity relationship (SAR) data informed our second round of compound synthesis. Key biological findings: it is possible to retain anti-invasion efficacy while having minimal effect upon cell growth inhibition (compound 22). Additionally, reduction of the C2-C3 double bond does not confer loss of

activity (compound 22) and appears to reduce off-target cell toxicity. Other findings: moving the C4 carbonyl group to generate the coumarin core confers loss of activity (consider compounds 23, 24 and 25).

Supplementary Figure 1c. Synthetic round #3. Key findings: substitution of the C4´-hydroxyl group with a halide is associated with maintenance of activity (compounds 37 and 38). We have identified a new chemical entity with potent anti-invasive effects, but which still retains growth inhibitory effects (compound 38).

Supplementary Figure 1d. Synthetic round #4. Key findings: we have identified a new chemical entity, now termed KBU2046 (compound 46), with anti-invasive efficacy at least equal to that of our starting compound, 4´,5,7-trihydroxyisoflavone, but that has no growth inhibitory effects. Compared to 4´,5,7-trihydroxyisoflavone, KBU2046 is non-planar, lacks hydroxyl groups, and particularly those that mediate ER binding, is halogen-substituted, and has a distinctly different biological profile. These characteristics place KBU2046 in a chemically distinct class, compared to our starting compound. Further, KBU2046 possesses novel biological characteristics; described below.

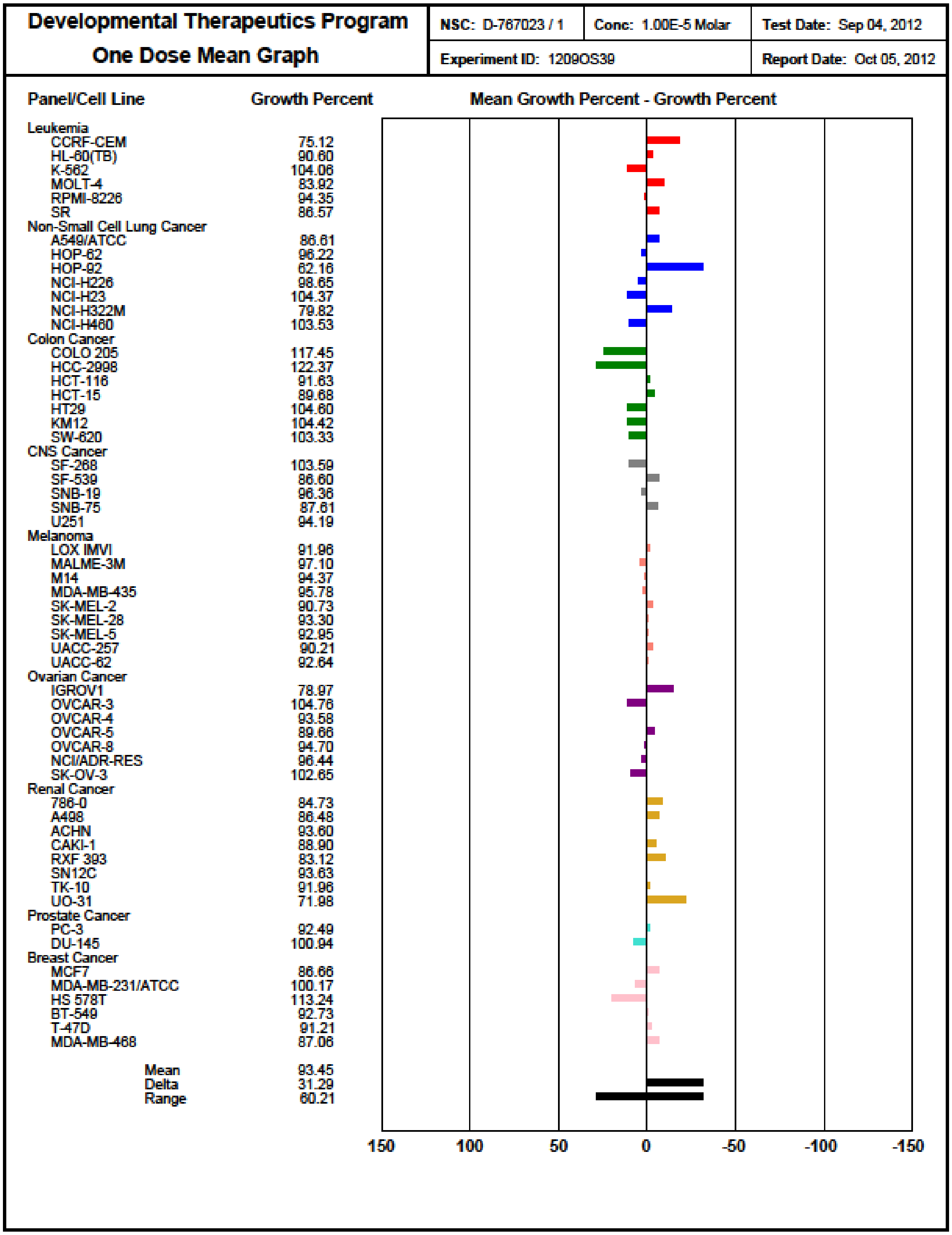

Supplementary Figure 2. KBU2046 has minimal-to-no cell toxicity in the NCI 60 cell line

panel. KBU2046 was submitted to the Developmental Therapeutics Program (DTP) of the US National Cancer Institute (NCI), underwent initial screening across the NCI 60 cell line panel per DTP protocol [1], and the resultant COMPARE diagram is depicted above. Based upon its lack of cell toxicity, NCI did not select KBU2046 to go on to multi-dose testing.

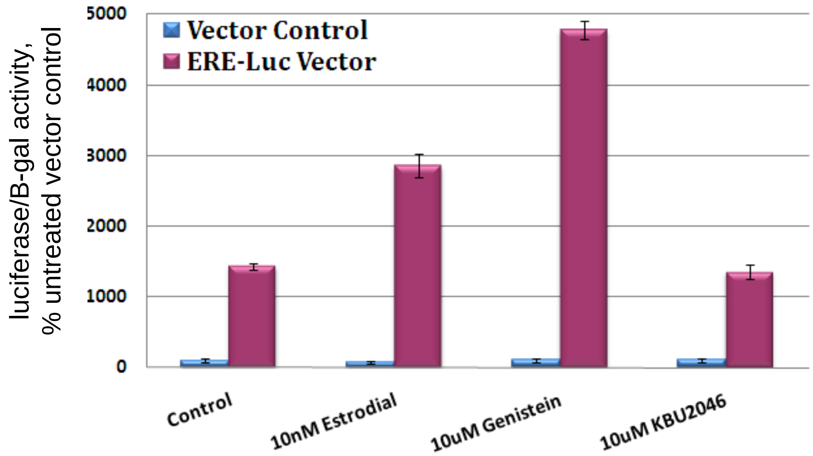

Supplementary Figure 3. KBU2046 does not activate the estrogen receptor (ER). Estrogen receptor positive MCF-7 cells were cultured under hormone free conditions transfected pERE-Luc or empty control vector, along with constitutive active β-gal, grown under estrogen-free conditions, and pre-treated for 24 hours with *nanomolar* concentrations of estradiol, or with *micromolar* concentrations of genistein or KBU2046, as indicated. Luciferase activity was measured, normalized to that of β-gal, and values expressed as the percent of untreated vector control cells. Values are the mean±SD of a single experiment, with similar results seen in a separate repeat experiment, both in replicates of N=2.

Chemical properties of KBU2046 that are associated with favorable pharmacokinetics

chemical property	molecular weight	calculated LogP	H-bond donor*	H-bond acceptor
recognized as favorable	<500	<5	</= 5	</= 10
KBU2046	242	3.65	0	2

*H-bond: hydrogen bond

Supplementary Figure 4. Chemical properties of KBU2046 that favor its ability to reach the cellular target when delivered systemically. In order for small compound probes to exert biological efficacy at the systemic level, they must be able to reach their protein target inside the body, and thus they must possess a favorable pharmacologic profile. Recognized chemical properties associated with favorable pharmacologic attributes [2] are provided in the table, as are the associated chemical properties of KBU2046.

a

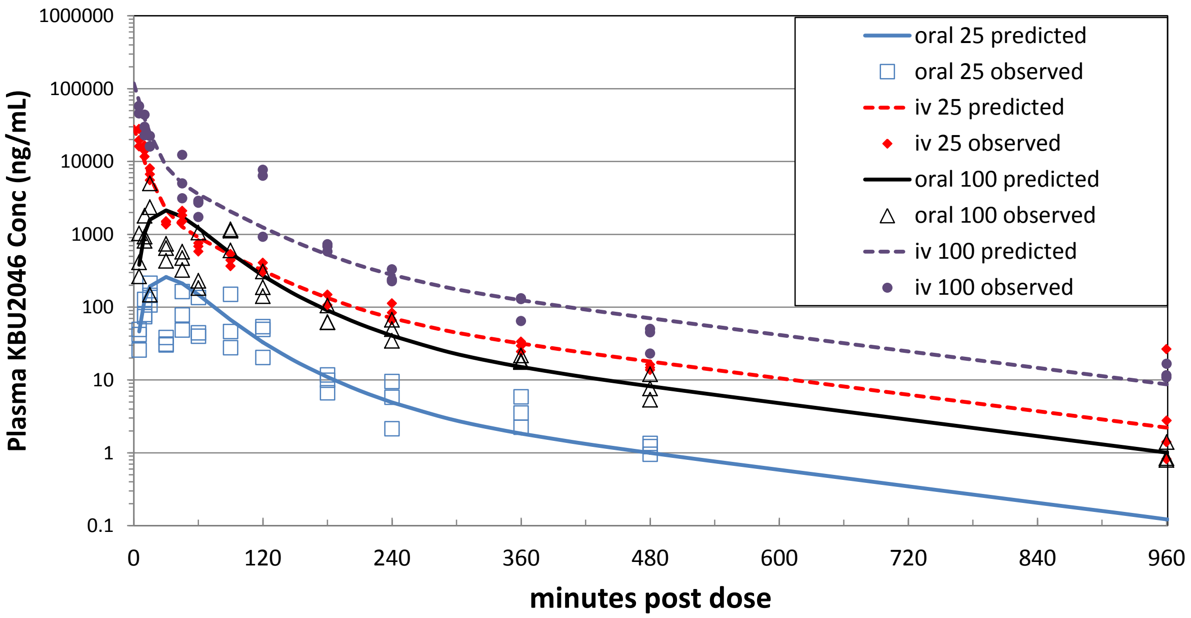

b

Pharmacokinetics of KBU2046 – Combined fit of both doses by both routes

Absorption delay (min)	32.3		
Distribution Volumes (mL)	Central	V_C	19.3
	Fast	V_F	20.4
	Slow	V_S	23.9
	Total	V_{SS}	63.6
Clearances (mL/min)	Fast	CL_F	0.61
	Slow	CL_S	0.11
	Elimination	CL_E	1.53
Elim Half-life (Hr)	2.27		
F(%)	25 mg/kg*	4.6	
	100 mg/kg*	10.1	

*by oral gavage

Supplementary Figure 5. Extensive pharmacokinetic (PK) analysis of KBU2046. Female CD1 mice were dosed with 25 or 100 mg KBU2046/kg via oral gavage or intravenous injection (iv), and blood collected at 0 (pre-dose), 5, 10, 15, 30, 45, 60, 90, 120, 180, 240, 360, 480 and

960 minutes post dose. For each route and time point, N=3 mice were sampled. Mice were only sampled once. (**a**) Concentration versus time plot. Individual data points are the resultant plasma concentrations from individual mice, are the mean of N=2 measurements, and are expressed as ng/ml. The 100 mg dosing data was re-plotted and expressed as nM, and constitutes manuscript Fig. 2c. (**b**) The resultant pharmacokinetic parameters. Values are parameter estimates from a naïve pooled data approach in which single plasma concentrations measured for individual animals were pooled for both routes of administration of both doses and modeled simultaneously, as described in Methods. Note that estimates of parameter variability are not associated with this approach [3], and are thus not reported here.

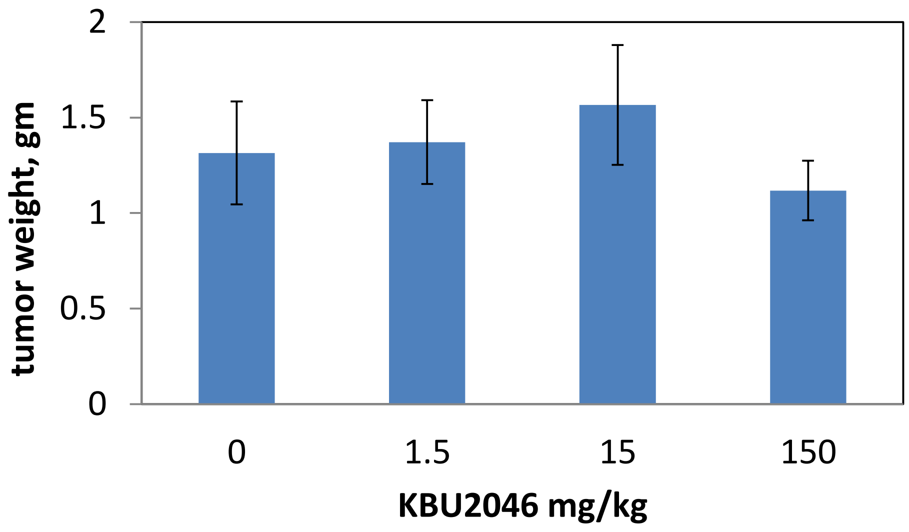

Supplementary Figure 6. KBU2046 does not inhibit primary tumor cell growth. Data are the mean±SEM tumor weight of mice treated in manuscript Figure 2a.

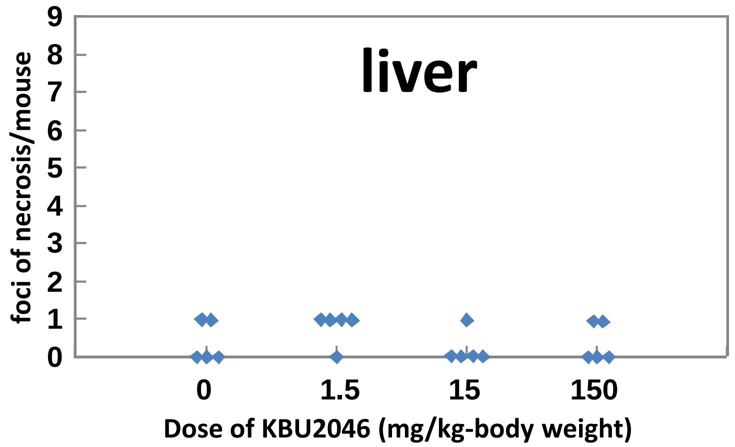

Supplementary Figure 7. KBU2046 treatment is not associated with systemic off-target effects. For histologic examination of tissue, cohorts of N=5 male 6-8 week old Balb/c athymic mice (Charles River Laboratories), which did not receive orthotopic implants, were treated with KBU2046 as described in manuscript Figure 2a. After 35 days of treatment, the following organs were harvested at necropsy, and stained with H&E (alternative staining methods as indicated): heart, lungs, esophagus, stomach, colon, small intestine, liver (Trichrome staining), kidneys (Trichrome staining), adrenals, bladder, prostate, spleen, pancreas, brain, testes, and bone marrow and peripheral blood (Giemsa staining). Organs were examined for damage using a semi-quantitative histological scoring system, as described [4]. No organ damage was observed, except in the livers of both control and treatment mice. Changes in the liver observed in control mice were not increased by KBU2046 treatment. Note that mice were immunocompromised, and that changes in the liver reflected episodic and minor foci of necrosis, consistent with a prior resolved infection; clinically, mice were all healthy. The graph above depicts the scoring of liver lesions, with each data point representing a single mouse.

For examination of organ function, studies used cohorts of 22-24 gm N=3 female and N=3 male (i.e., N=6 mice total per dose cohort) CD1 (ICR) mice (Charles River). Note that for 22-24

gm/mouse, this translated to 5-7 week old females and 4.5-5.5 week old males. Mice were dosed once intravenously with KBU2046 at 0 (vehicle), 15, 75 or 125 mg/kg-body weight. On day 8 and 14, all critical organs were weighed, and the following parameters measured in blood: cholesterol, triglycerides, alanine aminotransferase (ALT), aspartate aminotransferase (AST), total bilirubin, glucose, phosphorus, total protein, calcium, blood urea nitrogen (BUN), creatinine, albumin, Na, K, Cl, white blood cells (with differential), red blood cells, hemoglobin, platelets. No abnormal alterations in any of these parameters were observed, and there were no significant differences between treatment and control mice (data not shown). Immediate death was observed for mice dosed with 150 mg/kg IV.

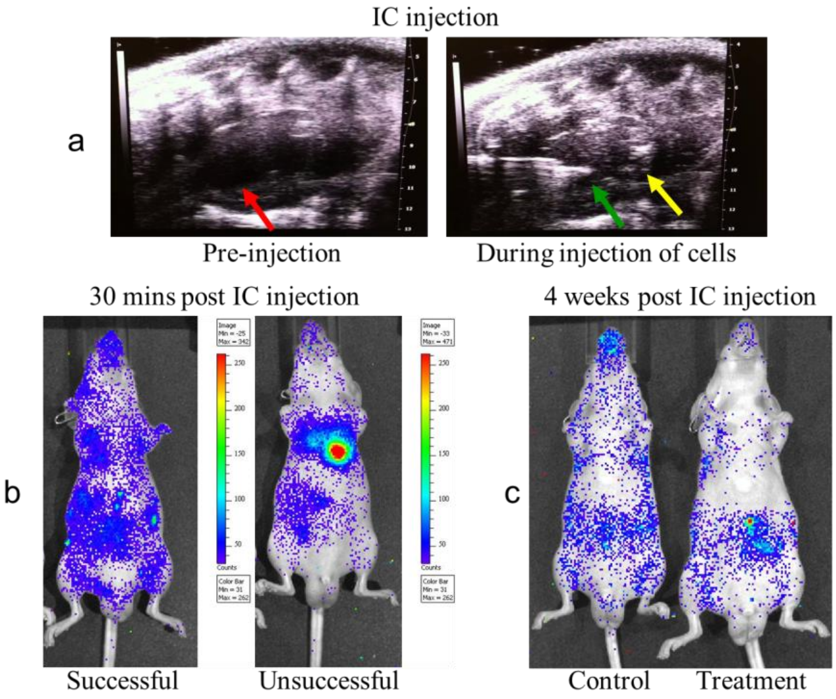

Supplementary Figure 8. Intra-cardiac injection of PC3-luc cells. (**a**) PC3-luc cells were injected under ultrasound guidance into the left ventricle. Depicted are snapshots of real time ultrasound images of a mouse undergoing intra-cardiac (IC) injection. The mouse is positioned

with the head to the right and the sternum on top. Red arrow: left ventricle; green arrow: the needle positioned within the left ventricle; yellow arrow: injectate containing PC3-luc cells exiting the ventricle through the aorta. (**b**) Confirmation of successful injection. Mice under IVIS imaging 30 minutes post IC injection. Successful injections are characterized by a relatively uniform distribution throughout the body, consistent with cells being injected into and distributed by the vasculature. Unsuccessful injections lack this pattern and are characterized by a local collection of cells in the thoracic cavity. (**c**) Representative IVIS images. Depicted are IVIS images from a control (non-treated) mouse, and from one treated with KBU2046 (i.e., treatment began 3 days prior to IC injection).

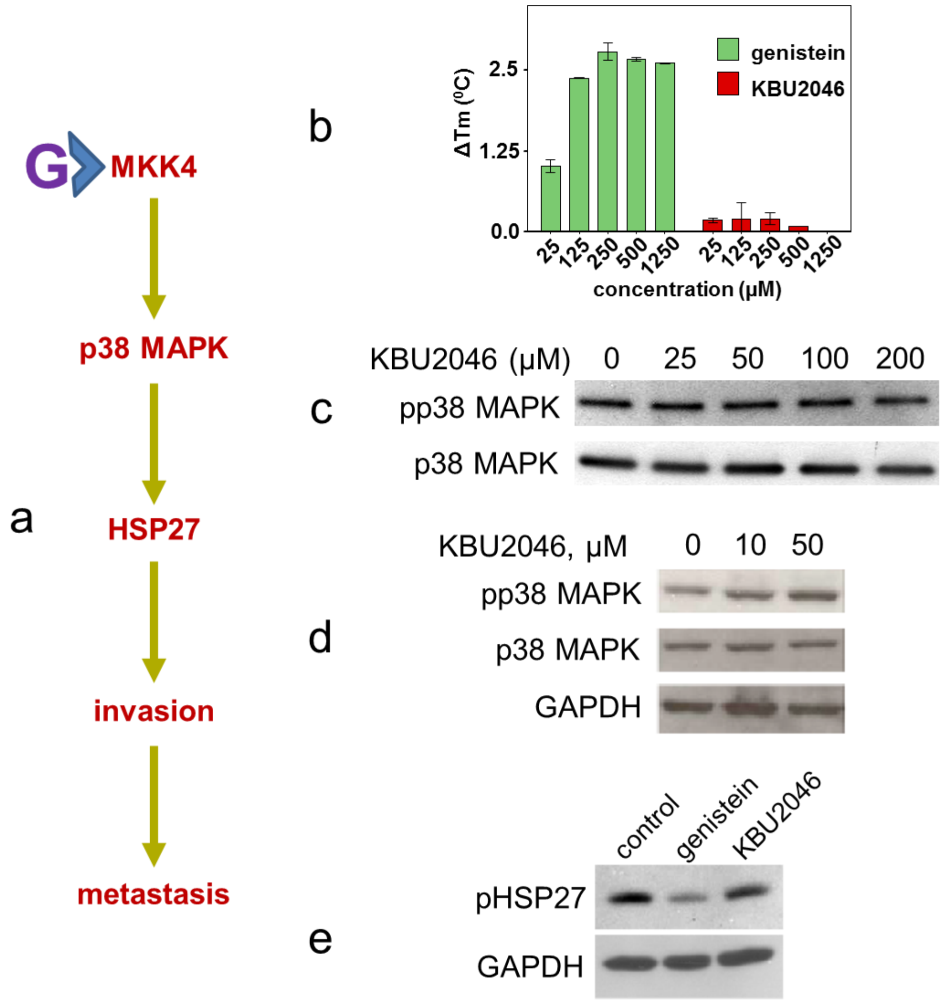

Supplementary Figure 9. KBU2046 does not inhibit the MKK4 pathway. (**a**) Depiction of established MKK4 pathway regulating human PCa cell metastasis. (**b**) KBU2046 does not bind to MKK4, as measured by fluorescence-based thermal shift assay. Values are the mean±SD (of N=2 replicates) increase in melting temperature (ΔTm) of purified recombinant MKK4 induced by the indicated concentrations of KBU2046 or genistein. (**c**) KBU2046 does not inhibit MKK4 in an *in vitro* kinase assay. The indicated concentrations of KBU2046 were added to recombinant activated MKK4, and its ability to phosphorylate kinase dead p38α MAPK (K53A) was measured by Western blot for total (p38 MAPK) and phosphorylated (pp38 MAPK) forms of p38 MAPK. (**d,e**) KBU2046 does not inhibit downstream phosphorylation of p38 MAPK or of HSP27 in cells. PC3-M cells were pre-treated for 24 hours with 50 μM KBU2046 or genistein, as indicated, then with TGFβ, and Western blot performed. All findings are from a single experiment, with similar findings in identical separate experiments, repeated at least once.

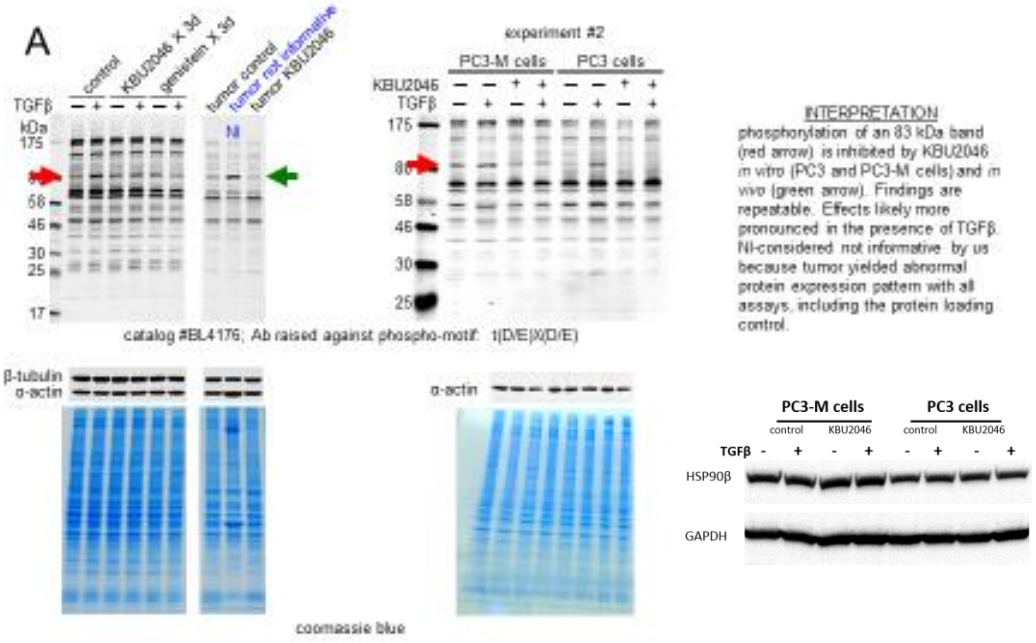

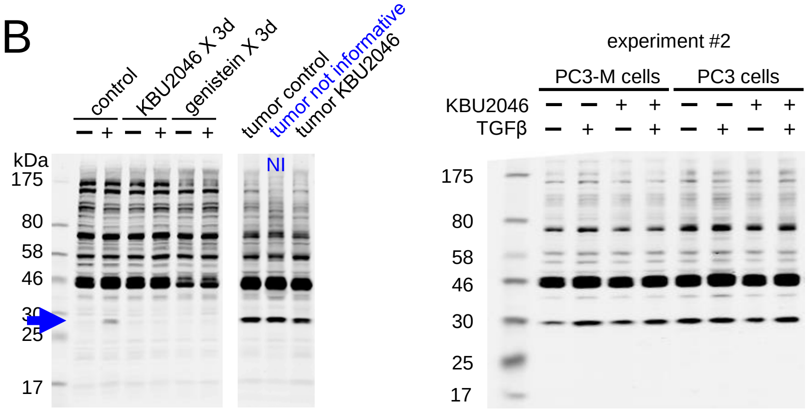

catalog #9614; RXX(s/t)

INTERPRETATION
phosphorylation of 27 kDa band (blue arrow) was inhibited by KBU2046 in experiment #1, but was not repeatable.

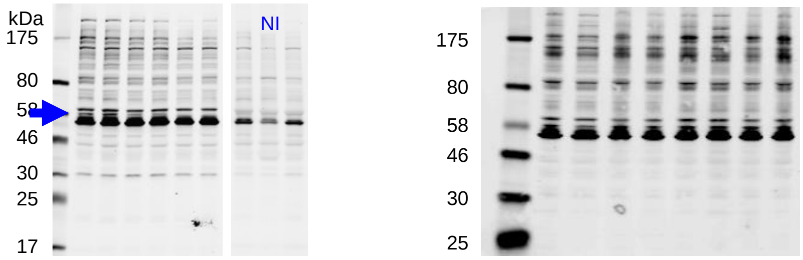

catalog #2325; PXsP

INTERPRETATION
phosphorylation of 58 kDa band (blue arrow) was inhibited by KBU2046 in experiment #1, but was not repeatable.

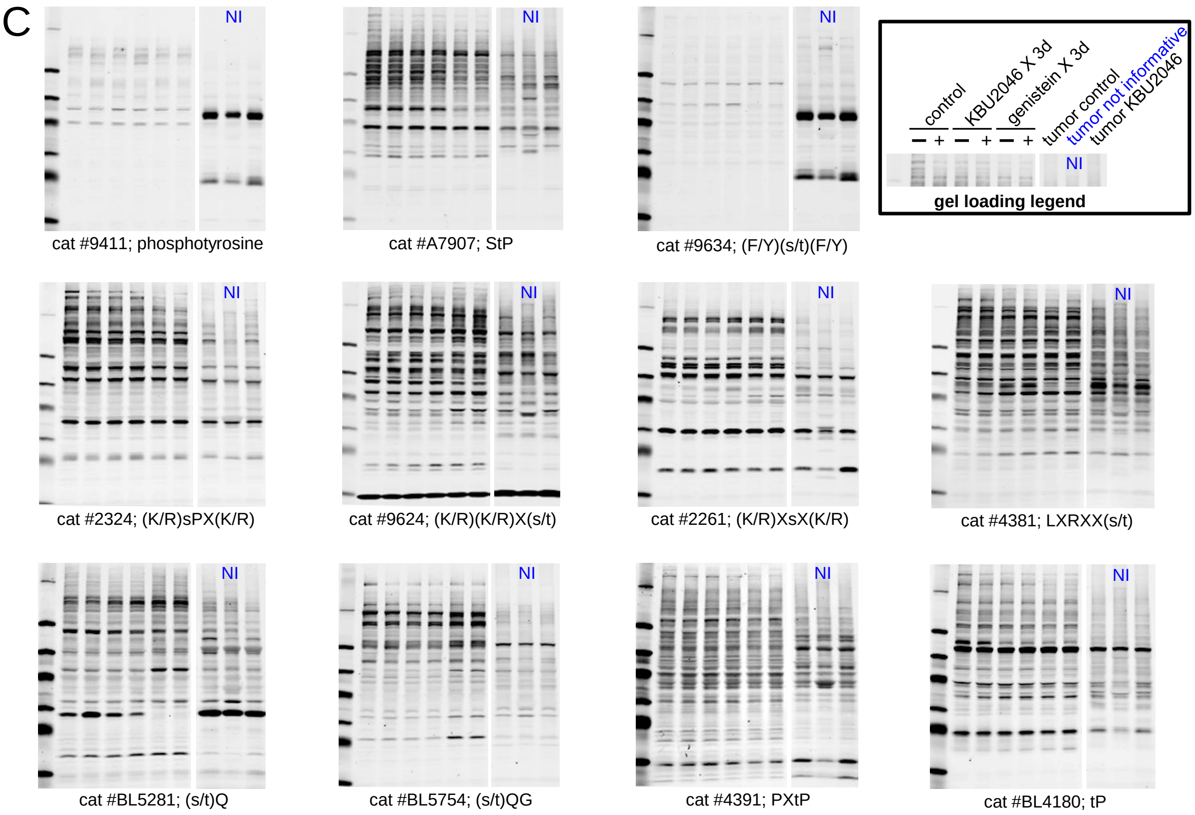

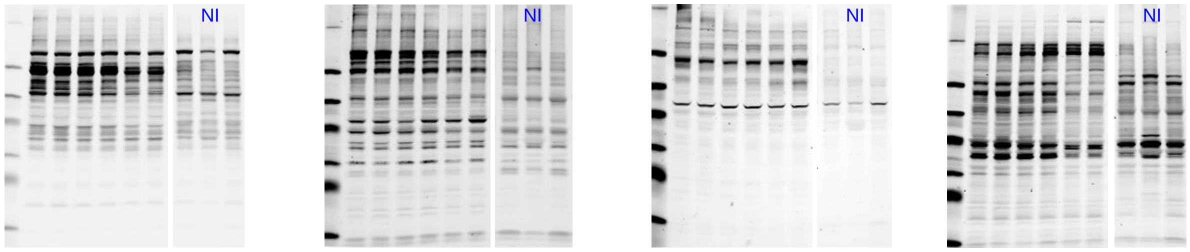

Supplementary Figure 10. Proteomic analysis of the effects of KBU2046 on the kinome. (**a-c**) Screening for effects on the kinome. PC3-M cells were pre-treated with 10 µM KBU2046, genistein or vehicle control for 3 days, then with ± TGFβ, as indicated. Resultant cell lysate, as well as lysate from tumors from mice treated with 150 mg/kg KBU2046 or control mice (from manuscript Figure 2a), were then probed with the KinomeView® panel of antibodies by Western blot. In instances where KBU2046 was inhibiting protein phosphorylation in cells and in tumors, a repeat experiment of PC3-M cells was conducted (Experiment #2). In addition to including PC3-M cells, as in Experiment #1, Experiment #2 expanded to examine effects on PC3 cells. (**a**) The identification of an 83 kDa band of interest (red arrows). This constitutes the only change that was repeatable across two experiments, and it was observed in PC3 and PC3-M cells, as well as in tumor tissue. (**b**) Bands of initial potential interest that did not repeat (blue arrows). (**c**) All other Western blots of phospho-motif antibodies that were evaluated on initial screen. NI = tumor not informative; this denotes a tumor sample that yielded an abnormal coomassie blue staining pattern. Data from this sample was therefore not considered further.

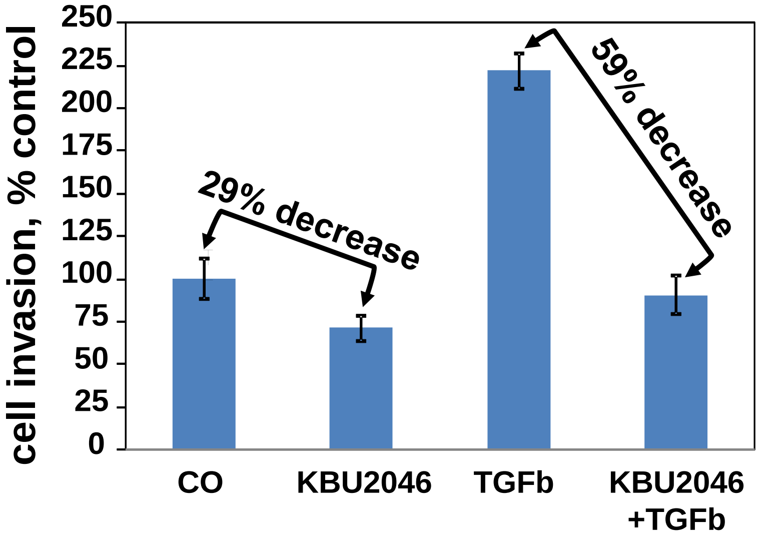

Supplementary Figure 11. KBU2046 retains efficacy even under conditions of TGFβ-stimulated increases in cell invasion. The invasion of PC3-M cells pretreated with KBU2046 or vehicle control (CO) and then with ± TGFβ was measured. Data are the mean±SEM of a single experiment; similar findings were observed in a replicate experiment (both experiments were in replicates of N=4).

A

Proteins exhibiting decreased expression with KBU2046

Protein	Control	KBU2046	fold change	MW, kd	Peptide	Protein Type
PGRMC2	515487	203250	-2.5	24	LLKPGEEPS*EYT*DEEDTKDHNK	Receptor, channel, transporter or cell surface protein
NudC	725332	291381	-2.5	38	NGSLDSPGKQDT*EEDEEEDEKDKGK	Cytoskeletal protein
CLASP2	1467931	606085	-2.4	141	SRS*DIDVNAAAGAK	Cytoskeletal protein
UBL5	229588	78757	-2.9	9	CNT*DDTIGDLKK	ubiquitin-like modifier
IWS1	221377	69188	-3.2	92	GHHVT*DSENDEPLNLNAS*DSESEELHR	Transcriptional regulator
SEC62	2261192	870178	-2.6	46	VGPGNHGTEGSGGERHS*DT*DSDRR	Receptor, channel, transporter or cell surface protein
SEC62	596485	213228	-2.8	46	VGPGNHGTEGS*GGERHS*DTDS*DRR	Receptor, channel, transporter or cell surface protein
RBM5	1208222	449457	-2.7	92	SEDGYHS*DGDYGEHDYR	RNA processing
BAT2D1	2243685	846172	-2.7	317	S*ES*SDFEVVPK	Cell cycle regulation
PIMT	568541	187146	-3.0	97	DRPHASGT*DGDES*EEDPPEHKPSK	Transcriptional regulator
FIP1L1	2756235	949876	-2.9	67	ERDHS*PTPSVFNS*DEER	RNA processing
NIN	917004	293104	-3.1	243	HVLS*DLEDDEVR	Cytoskeletal protein
VAMP1	332827	93333	-3.6	13	DQKLS*ELDDR	Unknown function
PITSLRE	2121720	647064	-3.3	93	DLLS*DLQDIS*DSER	Protein kinase, Ser/Thr (non-receptor)
LEO1	26743650	7926190	-3.4	75	MQNT*DDEERPQLS*DDER	Transcriptional regulator
PIMT	330713	70551	-4.7	97	DRPHAS*GT*DGDES*EEDPPEHKPSK	Transcriptional regulator
SSB	1591561	356289	-4.5	47	TKFAS*DDEHDEHDENGATGPVK	Transcriptional regulator
HSP90B	1356930	204324	-6.6	83	EIS*DDEAEEEKGEKEEEDKDDEEKPK	Chaperone
MFAP1	1970280	90214	-21.8	52	AALDALNT*DDENDEEEYEAWK	Unknown function

B

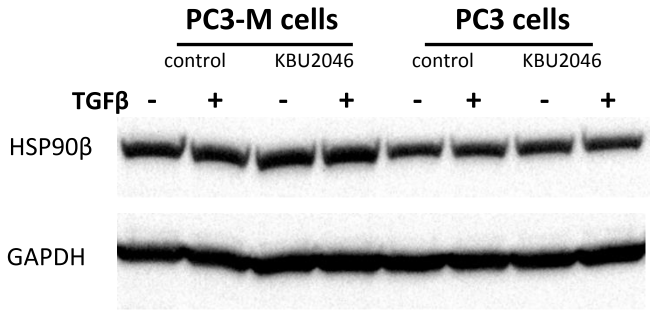

Supplementary Figure 12. Identification of the 83 kDa band using a proteomic approach.

(**a**) PC3 cells were pre-treated with 10 µM KBU2046 or vehicle (control) for 3 days, then treated with TGFβ for 1 hr, and the resultant cell lysates were subjected to immunoprecipitation with BL4176 (Kinoview® phospho-motif antibody). Proteins bound in this manner were analyzed in replicates of N=2 using PhosphoScan™ technology. This identified 483 unique phosphopeptide assignments from 306 parent proteins, with a mean false discovery rate of 0.30% (estimated via Sorcerer search of composite human database of forward and reverse protein entries). We only considered proteins where the average values for treatment and control were each 3 times that of background. Further, we required each of the N=2 replicate values (used to calculate the average) to be >/= 2.5 fold above background. We were only interested in situations in which protein decreased with KBU2046, and we required that decrease to be >/= 2.5 fold, compared to control. According to these parameters, there were 19 phospho-proteins whose expression decreased in cells treated with KBU2046, compared to control. These are depicted above. Recognizing that each of these proteins may have value, we elected to remain focused on the 83 kDa protein, and therefore only considered proteins +/- 5 kDa of this value. Only heat shock protein 90 (HSP90)β

met this criteria. (**b**) Evaluation of HSP90β levels. In order to assess whether phosphorylation changes detected in supplementary fig 11a were not due to changes in HSP90β protein, total HSP90β protein levels were measured by Western blot, after treatment of PC3 and PC3-M cells as described in fig 11a.

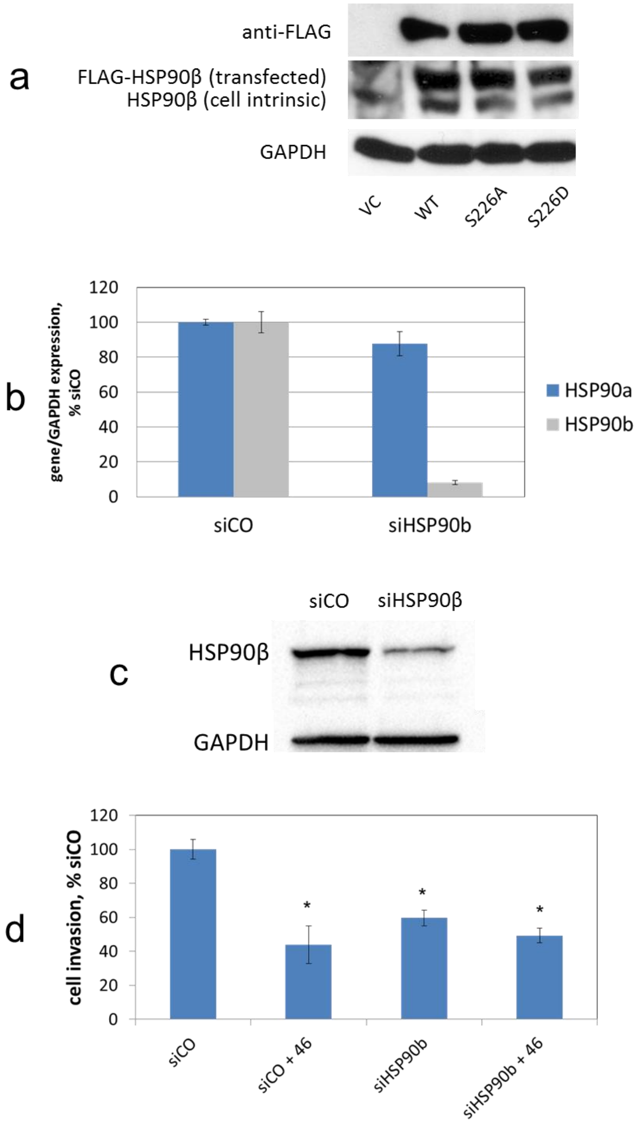

Supplementary Figure 13. Changes in HSP90β Ser^{226} structure as well as HSP90β expression regulate PCa cell invasion and KBU2046 efficacy. (**a**) Levels of HSP90β expression after transfection of cells. In manuscript Figs 4c and d, PC3-M cells were transfected with S226A-, S226D-, or WT-HSP90β, or empty vector (VC) and resultant effects upon cell invasion and KBU2046 efficacy measured. Depicted above are associated Western blots probing for HSP90β, FLAG (transfected HSP90β was FLAG-tagged) and GAPDH. (**b-d**) Knockdown of HSP90β decreases cell invasion and abrogates KBU2046 efficacy. PC3-M cells were transfected with siRNA to HSP90β (siHSP90b) or non-targeting siRNA (siCO). (**b**) The level of HSP90β (HSP90b) and HSP90α (HSP90a) transcript levels were measured by qRT/PCR, and expressed relative to that of GAPDH. (**c**) The level of HSP90β protein expression was measured by Western blot. (**d**) Cells were treated with KBU2046 (46) or vehicle control, and cell invasion measured. Values are the mean±SEM of a representative experiment of multiple experiments (all in replicates of N=3). *denotes t-test P value </= 0.05 compared to siCO.

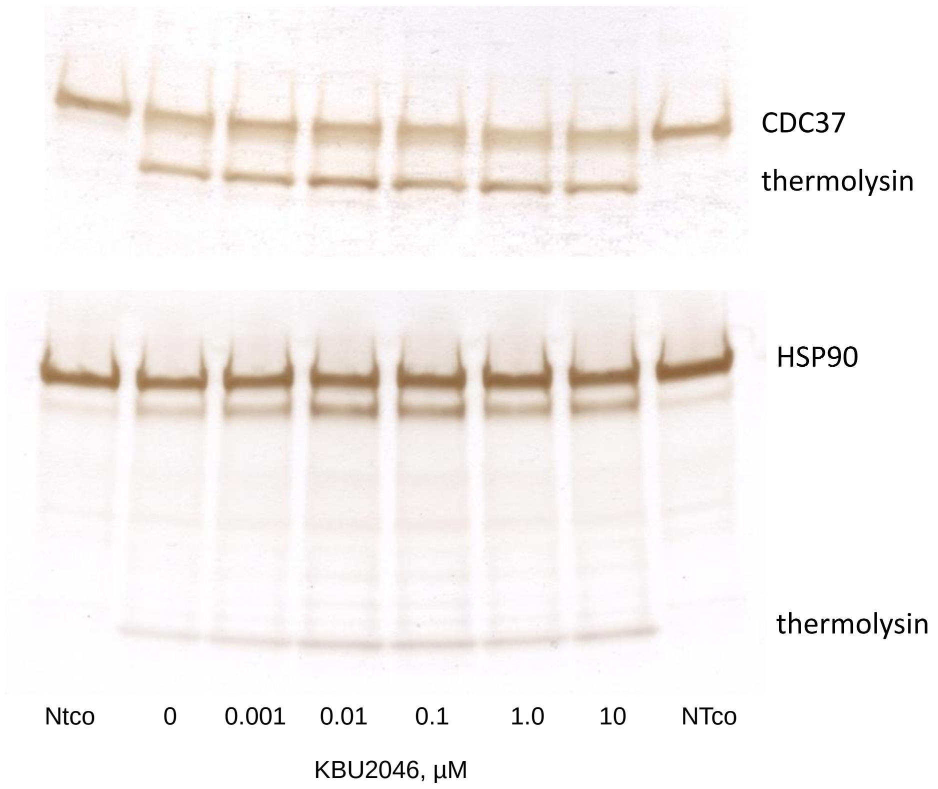

Supplementary Figure 14. KBU2046 does not bind HSP90β or CDC37. Studies used purified recombinant HSP90β or CDC37. Fluorescent thermal shift [5], isothermal titration calorimetry [6], and biolayer interferometry assays [7], were performed as described by us, and failed to provide evidence of KBU2046 binding to either HSP90β or CDC37 (data not shown). For DARTS assay, HSP90β or CDC37 were individually pre-incubated with KBU2046, thermolysin added, and reaction products were separated by SDS PAGE and visualized by silver stain (depicted above). NTco – no thermolysin control. However, when the DARTS assay was conducted with both HSP90β and CDC37 present, KBU2046 protected both proteins from degradation in a concentration-dependent manner (see manuscript Fig 5A).

a

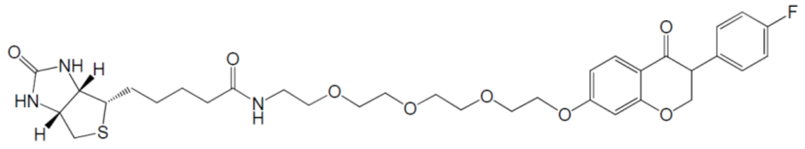

b

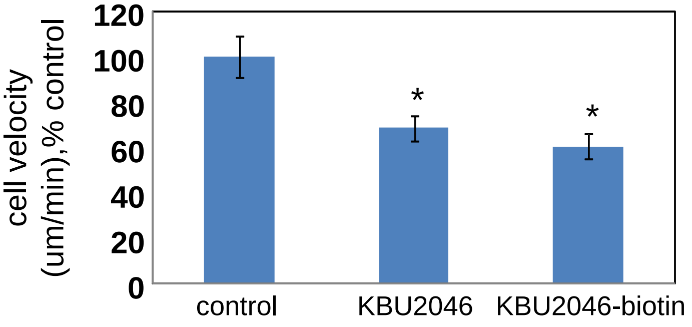

c

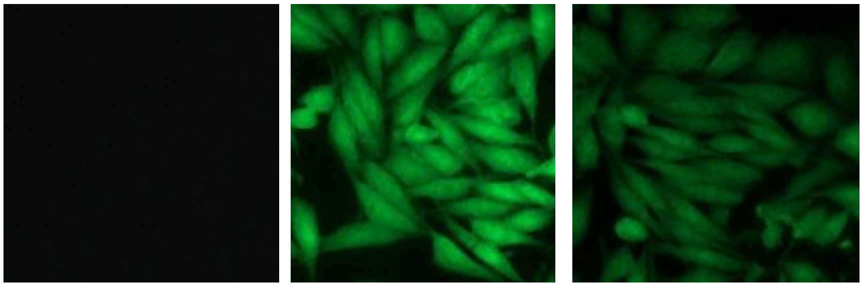

d

Signal intensity of KBU2046-biotin binding to protein microarray

		free KBU2046 absent		free KBU2046 present		% inhibition by competitor	
uniprot ID	name	0.5 uM	10 uM	0.5 uM	10 uM	0.5 uM	10 uM
P26440	Isovaleryl-CoA dehydrogenase	24040	55209	4832	48376	80%	12%
P78347	General transcription factor II-I	10702	62190	3878	35455	64%	43%
P21291	cysteine and glycine-rich protein 1	5643	21624	1158	14580	79%	33%

Supplementary Figure 15. KBU2046 binds to intact cells, but not to isolated proteins. (**a**) Chemical structure of KBU2046 linked to biotin (KBU2046-biotin) synthesized by us. (**b**) KBU2046-biotin is biologically active. PC3-M cells were pre-treated with 10 µM KBU2046 or with KBU2046-biotin for three days, and single cell motility assays conducted. Data are the mean±SEM of a single experiment of N $\geq$24 cells; * denotes Student's 2-sided t-test p-value </= 0.05 compared to control. (**c**) KBU2046-biotin labels permeablized cells in a manner that can be competed off. PC3-M cells were labeled with 1 µM KBU2046-biotin +/- 10 µM free KBU2046,

followed by detection with FITC-streptavidin, and visualization by fluorescent microscopy (with equal exposure times). (**d**) Protein array hybridization. KBU2046-biotin was hybridized to ProtoArray® Human Protein Microarray's at 0.5 and 10 µM with and without 10 fold excess free KBU2046. We first sought proteins that met the following criteria, and did so at both 0.5 and 10 µM concentrations of KBU2046-biotin (in the absence of free KBU2046): Z-Score greater than 2.5, Z-Factor greater than 0.5, CI P-value less than 0.05, negative control value <2,000 (relative fluorescence units; RFUs), and a signal/negative control signal of >10 and >5 for 10 µM and 0.5 µM conditions, respectively. This yielded 3 proteins, shown in table, for an initial hit rate of 0.03%. We then required free KBU2046 to inhibit binding by >75%. Of the two remaining candidates, we eliminated isovaleryl-CoA dehydrogenase (ICD). Its binding signal doubled on going from 0.5 to 10, while percent competition by free KBU2046 markedly decreased; we suspected non-specific binding by the biotin-linker moiety. We deemed cysteine and glycine-rich protein 1 (CSRP1) our top priority protein, though its binding characteristics at 10 µM + free KBU2046 were similar to ICD, albeit not as extreme. We thus evaluated whether KBU2046-biotin would pull down recombinant CSRP1, and failed to find any evidence of such (data not shown).

There were two additional notable findings from protein array studies. First, the positive control used in protein arrays was staurosporine. Staurosporine is similar to genistein in that both are small compound natural products that are broad spectrum kinase inhibitors. In contrast to the lack of binding by KBU2046-biotin, staurosporine bound to 214 proteins at levels that were >/= 10 fold above that of background. The vast majority of these proteins were kinases. Second, both HSP90β and CDC37 were present on the protein arrays, and were not bound by KBU2046-biotin.

Supplementary Figure 16.

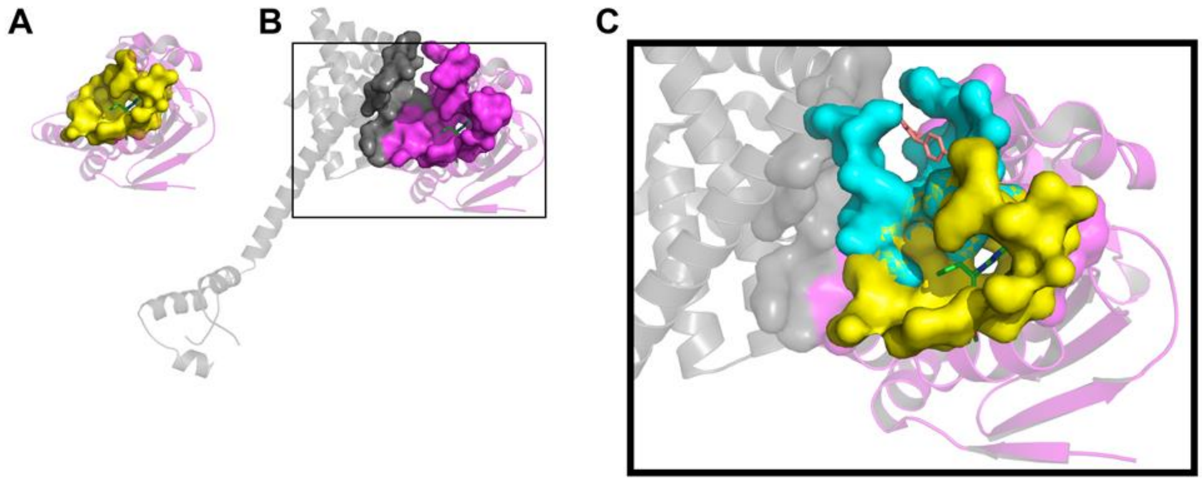

Supplementary Figure 16. **Structural model of HSP90β, CDC37 and KBU2046 interaction**. (**a**) The HSP90 (magenta) nucleotide binding site surface (A, yellow) shown with bound inhibitor [8]. When complexed with CDC37 (gray), a large cleft is formed at the interface (**b**). The CDC37 Arg167 residue dissects the cleft into two distinct sub-pockets (**c**). The nucleotide binding surface (C, yellow) is preserved, but a new sub-pocket (cyan) is formed. KBU2046 is shown docked into the newly formed site (C, wheat).

A marked feature of the HSP90 structure is the nucleotide binding site. The site, with solvent accessible area of 496.2 $Å^2$ and volume of 301.3 $Å^3$ [9], has been well-characterized and targeted by a variety of compounds for anti-cancer activity. In Supplementary Figure 16a, the site is shown in yellow surface representation, bound with a purine-based inhibitor (PDB id=1uym) [8]. When complexed with CDC37, an expansive surface, with solvent accessible area of 1446.4 $Å^2$ and volume of 2082.6 $Å^3$, is formed at the molecules' interface (Supplementary Figure 16b).

$Arg167_{cdc37}$ is drawn in to the nucleotide binding pocket and forms a hydrogen bond with the carboxyl side chain from $Glu33_{HSP90}$ [10]. It was shown that $Arg167_{cdc37}$ does not preclude access to the nucleotide binding site or displace any bound ligands [10]. It does, however, divide the large cleft into two distinct pockets: a newly formed pocket and the undisturbed, yet smaller, nucleotide binding site (Supplementary Figure 16c and manuscript Fig. 5b). The new pocket has solvent accessible area of 429.2 $Å^2$ and volume of 832.5 $Å^3$ and meets the criteria of a structural feature only present in the HSP90β-CDC37 complexed state.

The KBU2046 compound was docked into the newly formed pocket. A suite of docking software, representing different methodologies and approaches was applied (see High-Performance Molecular Modeling Platform in Methods). When allowed in the docking procedures, side chains from the HSP90β-CDC37 complex were allowed to be fully flexible. A consensus pose was reached with root mean square distance (RMSD) less than 1.1 Å over all atoms that exhibits no steric clashing with the complex. This model suggests that the molecule is capable of binding to this secondary site.

A dimer of the HSP90β structure in the closed conformation was modeled from *S. cerevisiae* HSP90A (PDB id=2cg9). In construction of the dimer, the HSP90β-CDC37 interface interactions were maintained. Position and orientation of the extended CDC37 regions were guided by cross-linking data that showed inter-domain cross-links between residues 53-347, 107-347, and 69-286 (manuscript Fig. 5d). This resulting structure shows agreement with other reported conformations [11]. In this model, both the ATP and proposed KBU2046 pockets remain intact in the dimerized complex.

LUMIER assay results

a

	Experiment # 1							Experiment # 2*								TGFbeta experiment						
	control		KBU2046		[Direction]			control		KBU2046		[Direction]			"X" IF t-test <0.05 AND same direction as Exp 1	control		KBU2046		[Direction]		"X" IF t-test <0.05 AND same
gene id	ave	stdev	ave	stdev	ratio 46/co	t-test	"X" IF t-test <0.05	ave	stdev	ave	stdev	ratio 46/co	t-test	"X" IF t-test <0.05		ave	stdev	ave	stdev	ratio 46/co	t-test	
ARAF	0.079	0.046	1.391	0.218	17.711	0.1434																
MAP3K15	4.330	0.123	3.982	0.069	0.920	0.0006	X	5.025	0.148	4.805	0.027	0.956	0.0115	X	X	4.928	0.103	5.126	0.073	1.040	0.0080	
AKT3	0.509	0.291	0.618	0.255	1.215	0.5441																
MAP3K14	5.136	0.195	5.170	0.057	1.007	0.7194																
PKN2	5.729	0.438	6.193	0.477	1.081	0.1472																
AMHR2	3.409	1.484	3.858	0.099	1.132	0.4763																
FYN	3.084	0.224	3.256	0.185	1.056	0.1761																
RIPK1	3.458	0.112	3.128	0.048	0.905	0.0003	X	3.673	0.116	3.363	0.117	0.915	0.0029	X	X	3.858	0.095	3.691	0.111	0.957	0.0345	X
LIMK2	5.529	0.270	5.634	0.213	1.019	0.5101																
CDK9	4.965	0.381	4.942	0.371	0.995	0.9260																
ARAF	0.201	0.145	0.205	0.154	1.021	0.9696																
TIE1	4.673	0.203	4.655	4.492	0.996	0.1102																
CDC2L1	2.066	0.131	2.145	0.128	1.038	0.3658																
PSKH2	1.368	0.108	0.905	0.295	0.662	0.0048	X	4.345	0.243	4.415	0.076	1.016	0.5556									
PINK1	6.245	0.167	6.328	0.284	1.013	0.5871																
MAPK7	3.645	0.361	3.103	0.326	0.851	0.0376	X	5.233	0.203	5.249	0.144	1.003	0.8833									
TNK1	2.735	0.203	2.529	0.169	0.925	0.1200																
AXL	0.877	0.158	0.942	0.249	1.074	0.6342																
TNK2	2.695	0.238	2.528	0.419	0.938	0.4604																
FGFR3	3.279	0.355	3.108	0.200	0.948	0.3770																
CDC2L5	5.302	0.473	4.575	0.323	0.863	0.0218	X	9.303	0.541	8.489	0.607	0.912	0.0553			6.026	0.251	6.038	0.183	1.002	0.9284	
PFTK2	5.397	0.732	5.555	0.379	1.029	0.6799																
ITK	3.411	0.099	3.946	0.156	1.157	0.0002	X	#NUM!	#NUM!	#NUM!	#NUM!	#NUM!	#NUM!									
MAP3K8	1.962	0.253	2.165	0.473	1.103	0.4784																
DSTYK	2.779	0.286	2.729	0.335	0.982	0.8051																
FGFR1	2.370	0.260	2.238	0.233	0.944	0.4203																
PSKH1	4.602	0.343	5.923	1.189	1.287	0.0258	X	7.782	0.811	6.742	0.679	0.866	0.0740									
INSRR	2.730	0.210	2.808	0.151	1.028	0.5223																
IRAK2	0.542	0.208	0.489	0.103	0.903	0.6267																
RAGE	6.854	0.674	5.133	1.091	0.749	0.0171	X	9.607	0.477	9.817	0.592	1.022	0.5538									
PRKACB	1.747	0.720	1.267	0.610	0.725	0.2417																
FER	0.983	0.244	0.763	0.150	0.777	0.0900																
STK11	0.417	0.412	0.460	0.233	1.101	0.8465																
MAP3K9	1.256	0.134	1.195	0.157	0.951	0.5267																
TESK2	3.883	0.267	3.629	0.152	0.935	0.1011																
JAK3	#NUM!	#NUM!	#NUM!	#NUM!	#NUM!	#NUM!		#NUM!	#NUM!	#NUM!	#NUM!	#NUM!	#NUM!									
NPR2	3.094	0.284	2.892	0.176	0.935	0.2124																
MOS	3.115	0.188	2.932	0.324	0.941	0.3046																
HCK	1.165	0.158	1.413	0.175	1.213	0.0471	X	3.203	0.131	2.899	0.096	0.905	0.0031	X								
NEK11	2.623	0.320	2.856	0.283	1.089	0.2583																
GRK4	4.415	0.768	5.946	1.563	1.347	0.0849																
FASTK	1.750	0.160	2.169	0.268	1.240	0.0169	X	1.493	0.149	1.280	0.077	0.858	0.0220	X								
MYLK4	1.963	0.905	3.068	0.769	1.563	0.0710																
DYRK4	5.188	0.281	5.811	0.148	1.120	0.0024	X	7.951	0.343	7.074	0.187	0.890	0.0010	X								
RAF1	0.305	0.079	0.080	0.076	0.261	0.0062	X	5.522	0.167	4.895	0.398	0.886	0.0271	X	X	10.639	0.721	8.866	0.479	0.833	0.0018	X
FLT4	0.652	0.207	0.504	0.130	0.773	0.2116																
CAMK2G	1.697	0.210	1.738	0.183	1.024	0.7502																
MAPK4	2.079	0.125	2.450	0.171	1.179	0.0044	X	4.981	0.183	4.873	0.275	0.978	0.4880									
TSSK2	0.700	0.250	0.474	0.242	0.678	0.1856																
PRKY	2.665	0.270	2.572	0.324	0.965	0.6357																
KSR2	4.058	0.264	3.758	0.283	0.926	0.1217																
FASTK	3.976	0.253	3.494	0.157	0.879	0.0069	X	7.242	0.892	7.601	1.227	1.050	0.6105									
PRKAA1	2.569	0.626	2.757	0.189	1.073	0.5400																
WNK1	1.062	0.473	1.785	0.274	1.680	0.0182	X	2.778	0.359	2.821	0.286	1.015	0.8413									
EPHB6	0.584	0.257	0.831	0.127	1.424	0.0894																
EPHB1	1.451	0.202	1.636	0.107	1.127	0.1087																
NTRK1	0.920	0.041	1.585	0.228	1.723	0.0002	X	1.716	0.101	1.991	0.162	1.161	0.0121	X	X	2.113	0.219	2.343	0.122	1.109	0.0740	
RIPK5	1.253	0.251	1.230	0.212	0.982	0.8827																
ABL1	1.100	0.374	1.376	0.396	1.251	0.2905																
DDR2	1.621	0.079	2.032	0.121	1.254	0.0002	X	4.023	0.233	4.221	0.172	1.049	0.1638			3.338	0.155	3.622	0.203	1.085	0.0375	
ALS2CR7	9.510	1.077	7.881	0.296	0.829	0.0115	X	8.346	1.457	8.632	1.509	1.034	0.7678									
MATK	1.248	0.144	0.147	0.192	0.118	0.0059	X	0.626	0.225	0.608	0.169	0.971	0.8881			1.826	0.229	2.042	0.332	1.118	0.2644	
MAST2	5.944	0.191	6.162	0.240	1.037	0.1499																
ULK3	4.127	0.185	3.813	0.184	0.924	0.0272	X	3.762	0.727	3.865	0.508	1.027	0.8019									
EIF2AK4	2.144	0.320	2.027	0.154	0.945	0.4823																
MYLK3	0.754	0.092	0.929	0.198	1.232	0.1112																
DCLK2	2.281	0.359	2.772	0.172	1.215	0.0247	X	4.372	0.375	4.557	0.208	1.042	0.3635									
ACVR1C	3.917	0.059	3.951	0.162	1.009	0.6717																
FRK	4.365	0.172	1.415	0.223	0.324	0.0000	X	4.367	0.484	6.456	1.876	1.478	0.0425	X		7.243	0.803	7.456	0.533	1.029	0.6345	
MYO3B	3.592	0.248	3.377	0.086	0.940	0.1038																
DYRK1B	0.552	0.384	0.864	0.279	1.565	0.1801																
MAP3K6	3.886	0.112	4.226	0.171	1.087	0.0059	X	4.660	0.262	4.520	0.204	0.970	0.3745			3.172	0.163	3.196	0.114	1.008	0.7907	
ICK	8.156	0.281	8.126	0.685	0.996	0.9283																
IRAK2	1.438	0.116	1.462	0.237	1.016	0.8484																
ACVR1B	0.734	0.188	0.864	0.124	1.176	0.2353																
PTK2	1.054	0.109	1.188	0.082	1.127	0.0588																
PRKX	1.212	0.063	1.707	0.167	1.408	0.0003	X	3.758	0.396	3.292	0.188	0.876	0.0450	X								
GRK6	1.106	0.419	0.921	0.453	0.833	0.5223																
PRKR	4.158	0.506	3.694	0.241	0.888	0.1008																
WNK4	0.052	0.290	0.602	0.116	11.555	0.0043	X	2.253	0.077	1.771	0.304	0.786	0.0089	X								
CDK7	5.060	0.061	4.014	0.525	0.793	0.0022	X	4.055	0.813	4.703	0.393	1.160	0.1469									
STYK1	0.710	0.356	0.803	0.082	1.130	0.5869																
CLK3	0.954	0.675	1.729	0.454	1.812	0.0658																
NEK8	1.319	0.278	1.432	0.252	1.086	0.5170																
PRKCB	4.179	0.860	4.805	1.940	1.150	0.5282																
MERTK	3.897	0.218	5.381	1.121	1.381	0.0446	X	9.313	2.826	6.975	0.207	0.749	0.1023									
PCTK3	1.515	0.222	1.853	0.243	1.223	0.0511																
LCK	3.348	0.221	3.199	0.123	0.956	0.2261																
EPHB6	#NUM!	#NUM!	#NUM!	#NUM!	#NUM!	#NUM!		3.704	1.168	2.557	0.855	0.690	0.1939									
LYN	7.163	1.003	6.202	0.401	0.866	0.0819																
ERBB2	1.122	0.092	0.658	0.197	0.586	0.0014	X	1.465	0.110	1.252	0.201	0.855	0.0717			1.042	0.197	1.355	0.152	1.301	0.0224	
HIPK4	0.521	0.196	0.862	0.200	1.653	0.0263	X	0.870	0.098	0.954	0.108	1.096	0.2339									
GRK7	2.358	0.447	2.021	0.308	0.857	0.2022																
MAP3K5	1.118	0.153	1.144	0.119	1.023	0.7729																
MAP3K2	4.937	0.318	3.951	1.063	0.800	0.0820																
ALK	0.105	0.228	0.052	0.170	0.497	0.6895																
HCK	0.967	0.186	0.695	0.213	0.719	0.0637																
ACVR2B	1.729	0.209	1.956	0.154	1.131	0.0873																
NTRK3	2.769	0.277	3.434	0.154	1.240	0.0016	X	1.606	0.877	1.173	0.286	0.730	0.3239									
GSK3A	2.698	0.079	2.755	0.186	1.021	0.5456																
TSSK6	3.431	0.232	3.405	0.292	0.993	0.8903																

MAP2K7	3.508	0.128	3.571	0.067	1.018	0.4704																
CLK3	#NUM!	#NUM!	#NUM!	#NUM!	#NUM!	#NUM!		6.018	1.534	6.050	0.853	1.005	0.9712									
MAP2K5	1.934	0.406	2.167	0.495	1.121	0.4388																
EPHA2	1.086	0.187	1.224	0.079	1.127	0.1670																
TNNI3K	4.344	1.520	6.112	0.223	1.407	0.0329	X	1.613	0.690	2.387	0.783	1.480	0.1358									
CAMKK1	4.826	0.556	5.449	0.294	1.129	0.0576																
TESK1	3.101	0.161	3.283	0.087	1.059	0.0571																
DCAMKL2	2.790	0.552	1.775	0.455	0.636	0.0132	X	2.474	0.116	2.736	0.118	1.106	0.0074	X								
PDIK1L	0.824	0.072	0.823	0.176	0.998	0.9849																
DDR1	1.251	0.175	1.304	0.121	1.043	0.5905																
SGK2	3.330	0.272	3.389	0.410	1.018	0.7934																
SGK1	3.303	0.781	3.718	1.174	1.126	0.5650																
GSG2	1.295	0.082	0.829	0.121	0.640	0.0001	X	1.865	0.100	1.710	0.088	0.917	0.0310	X	X	2.297	0.196	2.594	0.099	1.129	0.0165	
ILK	1.378	0.367	1.476	0.223	1.071	0.6233																
SRPK3	0.213	0.076	0.144	0.365	0.676	0.6901																
EIF2AK4	1.854	0.441	2.836	0.272	1.530	0.0028	X	3.082	0.187	2.895	0.233	0.939	0.1980									
PKN1	3.576	0.684	6.517	2.140	1.823	0.0191	X	6.602	1.278	5.328	2.017	0.807	0.3075									
BTK	0.603	0.540	0.547	0.231	0.908	0.8373																
PRKG2	2.250	0.132	2.417	0.116	1.074	0.0663																
PRKCE	1.593	0.151	1.812	0.272	1.138	0.1538																
PLK2	3.573	1.245	1.739	0.493	0.487	0.0156	X	2.301	0.424	3.021	0.450	1.313	0.0315	X								
RIOK3	2.081	0.395	1.800	0.298	0.865	0.2407																
MAPK7	3.186	1.793	4.239	1.820	1.330	0.4142																
PRKACB	3.398	0.697	3.543	0.614	1.043	0.7090																
IKBKE	0.995	0.215	1.171	0.132	1.178	0.1553																
CDK10	1.443	0.145	1.410	0.244	0.977	0.8016																
TSSK1B	2.361	0.305	2.348	0.248	0.995	0.9451																
NUAK2	1.749	0.344	1.445	0.640	0.826	0.3777																
PIK3R4	6.260	1.819	6.290	1.580	1.005	0.9806																
CSF1R	1.660	0.318	1.586	0.471	0.956	0.7806																
STK32C	1.488	0.128	1.574	0.192	1.058	0.4297																
MYLK2	3.673	2.703	4.497	2.360	1.224	0.6399																
RIPK2	0.632	1.422	2.256	0.451	3.567	0.0410	X	1.095	0.272	1.434	0.153	1.309	0.0415	X	X	1.549	0.192	1.386	0.264	0.895	0.2959	
MAP4K4	3.507	0.308	3.132	0.410	0.893	0.1399																
FLT3	3.857	0.298	4.130	0.118	1.071	0.0926																
CAMKV	0.956	0.643	0.605	1.430	0.633	0.6305																
MAP3K12	4.742	0.452	4.439	0.399	0.936	0.2927																
MAPK15	2.412	0.483	2.686	0.337	1.113	0.3291																
NEK2	1.128	0.150	1.572	0.285	1.394	0.0150	X	1.247	0.275	2.113	0.133	1.694	0.0002	X	X	2.780	0.212	2.416	0.988	0.869	0.4433	
ERBB3	0.823	0.318	0.270	0.620	0.327	0.1133																
ITK	2.830	0.422	2.697	0.196	0.953	0.5405																
CSNK1A1	1.164	0.185	1.390	0.210	1.194	0.1086																
TYK2	2.450	0.154	2.392	0.385	0.976	0.7633																
CDK8	3.037	2.595	0.856	1.241	0.282	0.1497																
BLK	0.821	0.434	1.054	0.251	1.283	0.3300																
RET	0.477	0.135	0.586	0.105	1.229	0.1899																
CSNK1D	1.220	0.213	1.376	0.129	1.128	0.1992																
BMPR1A	2.117	0.473	2.129	0.553	1.006	0.9713																
NRBP2	0.967	0.346	0.686	0.226	0.709	0.1662																
LYN	1.455	0.192	1.188	0.078	0.817	0.0204	X	1.052	0.260	1.534	0.238	1.458	0.0158	X								
PIM3	1.736	0.489	1.465	0.423	0.844	0.3759																
CSNK1D	2.117	0.350	1.322	0.569	0.625	0.0288	X	0.718	0.490	1.630	0.094	2.270	0.0035	X								
STK33	1.076	0.079	1.193	0.111	1.109	0.0920																
RPS6KA2	1.881	0.310	1.399	0.469	0.744	0.0912																
ALPK1	4.723	0.435	4.461	0.760	0.945	0.5231																
PRKCQ	1.413	0.278	1.679	0.198	1.188	0.1202																
CDK6	3.962	1.444	0.504	0.476	0.127	0.0021	X	2.162	0.401	2.095	0.218	0.969	0.7507									
MUSK	0.045	0.214	0.289	0.173	6.453	0.0828																
CSNK1E	1.477	0.211	1.058	0.153	0.716	0.0070	X	1.202	0.203	1.414	0.287	1.176	0.2145									
LCK	2.463	0.238	1.989	0.120	0.807	0.0041	X	2.114	0.161	2.159	0.206	1.021	0.7116									
KSR	4.498	0.489	4.010	0.923	0.891	0.3264																
TP53RK	2.039	0.339	0.821	0.527	0.402	0.0024	X	1.116	1.217	0.554	0.338	0.496	0.1356									
PRKCG	1.274	0.421	1.023	0.212	0.803	0.2686																
PTK2B	1.197	0.232	0.953	0.228	0.796	0.1316																
MAP4K2	1.356	0.713	1.689	0.340	1.245	0.3735																
DKFZp761P0	6.119	0.491	6.961	0.340	1.138	0.0136	X	5.330	0.471	4.250	0.519	0.797	0.0087	X								
CDK3	2.700	0.262	1.900	0.361	0.704	0.0039	X	2.729	0.079	2.716	0.140	0.995	0.8594									
LOC653052	6.087	0.559	4.891	0.749	0.803	0.0210	X	5.173	0.401	4.899	0.531	0.947	0.3840									
PRKCI	0.432	0.241	0.380	0.153	0.879	0.6931																
PRKCH	1.849	0.564	1.926	0.501	1.041	0.8260																
FES	0.898	0.181	0.874	0.166	0.973	0.8324																
IKBKE	1.013	0.495	1.252	0.490	1.236	0.4645																
ADRBK2	4.031	0.583	3.763	0.391	0.934	0.4192																
RPS6KA4	0.922	0.091	0.963	0.152	1.044	0.6244																
PTK6	0.897	0.151	0.903	0.275	1.006	0.9691																
TGFBR2	1.451	0.430	1.339	0.509	0.923	0.7187																
RET	2.180	0.131	2.267	0.197	1.040	0.4361																
RIPK3	0.590	0.110	0.780	0.239	1.323	0.1433																
LOC91807	4.422	0.082	4.549	0.140	1.029	0.1173																
ACVRL1	1.584	0.129	1.917	0.104	1.210	0.0020	X	4.261	1.261	2.075	0.114	0.487	0.0057	X								
PRKCB1	3.747	0.403	3.161	0.245	0.844	0.0241	X	4.210	0.163	3.664	0.257	0.870	0.0039	X	X	3.602	0.168	3.739	0.140	1.038	0.2000	
SGK	5.826	1.810	4.060	0.685	0.697	0.0758																
RPS6KL1	3.798	0.174	4.075	0.239	1.073	0.0686																
EPHA10	1.057	0.109	1.049	0.146	0.992	0.9209																
TBK1	1.820	0.576	1.428	0.443	0.785	0.2627																
CCRK	3.659	0.214	3.712	0.363	1.014	0.7865																
TYRO3	2.921	0.234	2.858	0.485	0.978	0.7997																
JAK1	1.446	0.190	0.720	0.208	0.498	0.0004	X	0.238	0.364	0.895	0.261	3.757	0.0112									
TYRO3	1.103	0.477	0.695	0.340	0.630	0.1579																
CAMK1G	1.340	0.671	1.078	0.297	0.805	0.4480																
RPS6KA5	2.667	0.270	2.849	0.365	1.068	0.3949																
ADCK1	3.888	0.452	1.250	0.519	0.322	0.0001	X	1.692	0.212	1.692	0.199	1.000	0.9973									
TSSK1	3.612	0.592	2.788	0.447	0.772	0.0378	X	1.482	0.645	0.471	0.273	0.318	0.1572									
LIMK1	0.083	0.179	1.057	0.201	12.657	0.0024	X	0.574	0.197	1.011	0.128	1.762	0.0032	X	X	1.484	0.080	1.450	0.131	0.977	0.6355	
PRKACG	0.465	0.109	1.243	0.078	2.673	0.0000	X	0.675	0.172	0.957	0.156	1.418	0.0263	X	X	1.514	0.211	1.588	0.141	1.049	0.5348	
EPHB1	4.393	0.412	3.037	0.224	0.691	0.0002	X	4.125	0.137	4.004	0.149	0.971	0.2176									
DSTYK	#NUM!	#NUM!	#NUM!	#NUM!	#NUM!	#NUM!		#NUM!	#NUM!	#NUM!	#NUM!	#NUM!	#NUM!									
FGR	1.102	0.268	0.957	0.181	0.868	0.3456																
CDK10	1.125	0.909	1.989	0.204	1.768	0.0718																
PAK6	0.285	0.059	0.542	0.953	1.901	0.5642																
PRKCZ	0.920	1.061	1.747	0.659	1.898	0.2144																
ROR2	0.844	0.231	0.715	0.123	0.847	0.3026																
IRAK3	1.081	0.228	0.983	0.131	0.910	0.4294																
TSSK4	2.490	0.544	2.877	0.463	1.155	0.2610																
EPHA1	1.145	0.313	1.282	0.174	1.120	0.4167																
STK32A	2.214	0.623	2.645	0.845	1.195	0.3848																
IGF1R	0.499	0.159	0.669	0.272	1.340	0.2638																

DAPK3	#NUM!	#NUM!	#NUM!	#NUM!	#NUM!	#NUM!		#NUM!	#NUM!	#NUM!	#NUM!	#NUM!	#NUM!									
PDGFRB	1.001	0.134	0.864	0.102	0.863	0.1070																
CDK4	0.590	0.331	1.490	0.472	2.526	0.0482	X	0.386	0.116	0.620	0.135	1.606	0.0188	X	X	1.465	0.139	1.473	0.247	1.006	0.9482	
EPHA3	2.908	2.094	1.929	0.457	0.663	0.3962																
CAMK2B	1.553	0.275	1.121	0.355	0.722	0.0402	X	1.454	0.157	0.279	0.178	0.192	0.0154	X	X	1.572	0.201	1.490	0.159	0.948	0.4963	
BMX	0.235	0.142	0.244	0.164	1.037	0.9299																
PRKAA2	0.780	0.544	0.316	0.556	0.405	0.2190																
PFTK1	2.111	0.666	1.865	0.545	0.883	0.5396																
DYRK2	#NUM!	#NUM!	#NUM!	#NUM!	#NUM!	#NUM!		0.193	0.761	0.700	0.564	3.625	0.2869									
AURKC	2.364	0.151	2.182	0.325	0.923	0.2887																
MGC42105	#NUM!	#NUM!	#NUM!	#NUM!	#NUM!	#NUM!		#NUM!	#NUM!	#NUM!	#NUM!	#NUM!	#NUM!									
NTRK2	2.805	0.392	1.605	0.181	0.572	0.0003	X	0.644	0.448	1.284	0.613	1.996	0.0956									
CSNK1A1L	0.859	0.189	1.165	2.411	1.356	0.0769																
AKT1	2.796	0.486	2.986	0.417	1.068	0.5257																
UHMK1	3.527	0.294	2.343	0.336	0.664	0.0003	X	5.444	1.408	4.464	0.201	0.820	0.1621									
PASK	0.338	0.040	0.058	0.097	0.171	0.0003	X	1.003	0.082	1.003	0.052	1.000	0.9986									
PDPK1	1.336	0.281	1.370	0.493	1.025	0.8995																
PHKG2	1.488	0.300	0.873	0.779	0.587	0.1384																
EPHA4	0.753	0.260	0.837	0.162	1.112	0.5557																
ADRBK1	1.457	0.570	1.252	0.303	0.860	0.4988																
ZAK	1.100	2.801	0.525	2.338	0.477	0.6833																
PAK4	#NUM!	#NUM!	#NUM!	#NUM!	#NUM!	#NUM!		#NUM!	#NUM!	#NUM!	#NUM!	#NUM!	#NUM!									
LOC91461	2.065	0.409	1.266	0.252	0.613	0.0059	X	1.306	0.339	0.718	0.102	0.550	0.0004	X	X	0.243	0.605	0.482	0.611	1.982	0.5517	
STRADB	7.085	1.327	7.189	1.728	1.015	0.9211																
DAPK1	0.625	0.264	0.561	0.229	0.897	0.6909																
PLK3	0.547	0.861	0.590	0.630	1.079	0.9304																
PDGFRA	0.597	0.160	0.316	0.185	0.530	0.0332	X	0.541	0.070	0.706	0.074	1.305	0.0067	X								
TGFBR1	1.397	0.172	1.790	0.083	1.281	0.0017	X	0.649	0.293	1.140	0.183	1.755	0.0131	X	X	0.834	0.342	1.209	0.152	1.449	0.0558	
SGK3	3.409	0.840	4.290	0.129	1.258	0.0492	X	3.956	0.856	6.425	1.763	1.624	0.0226	X	X	3.680	0.669	4.991	0.420	1.356	**0.0060**	X
EIF2AK1	3.107	0.410	2.535	0.371	0.816	0.0496	X	5.260	1.047	4.500	0.453	0.855	0.1746									
MAP3K7	#NUM!	#NUM!	#NUM!	#NUM!	#NUM!	#NUM!		4.536	2.008	4.623	1.335	1.019	0.9533									
PRKCA	3.296	0.384	3.636	0.156	1.103	0.1045																
BMPR1B	3.233	0.993	2.259	0.777	0.699	0.1227																
PTK6	1.047	1.084	0.578	0.204	0.551	0.0703																
LOC340371	1.130	0.157	1.462	0.099	1.293	0.0040	X	0.843	0.158	1.251	0.208	1.483	0.0083	X	X	0.969	0.326	1.179	0.345	1.217	0.3516	
TRIB3	1.942	0.830	0.984	3.497	0.507	0.5675																
STK38L	2.380	0.634	1.941	0.399	0.816	0.2269																
ULK4	#NUM!	#NUM!	#NUM!	#NUM!	#NUM!	#NUM!		#NUM!	#NUM!	#NUM!	#NUM!	#NUM!	#NUM!									
SIK1	0.911	0.911	0.026	0.332	0.028	0.1938																
PCTK2	1.153	0.188	1.425	0.292	1.236	0.1179																
AURKB	1.221	0.258	1.233	0.108	1.009	0.9298																
GRK6	0.883	2.015	1.246	0.740	1.411	0.7178																
EGFR	1.393	0.048	1.386	0.047	0.995	0.8104																
MINK1	2.296	0.310	2.527	0.157	1.101	0.1756																
AKT2	2.631	0.258	1.776	0.261	0.675	0.0008	X	3.566	1.132	2.682	0.457	0.752	0.1443									
RPS6KA1	0.428	0.373	0.654	0.697	1.529	0.5395																
HIPK2	3.828	0.583	3.938	0.439	1.029	0.7462																
ULK2	2.118	0.141	1.955	0.294	0.923	0.2962																
SRPK1	3.743	1.101	3.603	0.469	0.963	0.8011																
SLK	2.951	0.983	3.136	0.581	1.063	0.7266																
BMPR2	2.438	0.366	2.617	0.666	1.074	0.6125																
PLK4	2.120	0.180	1.943	0.071	0.917	0.0760																
BCKDK	#NUM!	#NUM!	#NUM!	#NUM!	#NUM!	#NUM!		0.844	0.531	0.525	1.168	0.622	0.6314									
FASTKD1	5.727	1.674	4.793	1.125	0.837	0.4458																
MAP2K6	1.997	2.160	1.304	1.338	0.653	0.5878																
JAK2	#NUM!	#NUM!	#NUM!	#NUM!	#NUM!	#NUM!		1.835	1.730	2.070	1.035	1.128	0.3378									
LMTK2	1.188	0.729	0.813	0.671	0.684	0.4210																
AURKA	1.444	0.478	1.528	0.286	1.058	0.7435																
ACVR2A	3.715	1.032	3.522	1.019	0.948	0.7877																
DYRK2	#NUM!	#NUM!	#NUM!	#NUM!	#NUM!	#NUM!		#NUM!	#NUM!	#NUM!	#NUM!	#NUM!	#NUM!									
PAK2	0.967	1.814	0.374	0.862	0.387	0.3424																
MAP4K3	2.095	0.377	1.814	0.560	0.866	0.3791																
PRKD2	0.726	0.481	0.677	0.303	0.932	0.8513																
STK38	3.275	0.355	3.097	0.267	0.946	0.3983																
NEK10	#NUM!	#NUM!	#NUM!	#NUM!	#NUM!	#NUM!		0.927	0.864	1.017	1.374	1.097	0.9045									
PTK2	2.156	0.582	1.369	0.829	0.635	0.1204																
BRAF	#NUM!	#NUM!	#NUM!	#NUM!	#NUM!	#NUM!		#NUM!	#NUM!	#NUM!	#NUM!	#NUM!	#NUM!									
NEK9	1.092	0.237	0.910	0.185	0.833	0.2118																
CLK2	0.966	0.088	0.991	0.229	1.026	0.8270																
MGC16169	#NUM!	#NUM!	#NUM!	#NUM!	#NUM!	#NUM!		#NUM!	#NUM!	#NUM!	#NUM!	#NUM!	#NUM!									
CHEK1	0.094	0.844	1.232	0.620	13.128	0.4619																
PIM1	#NUM!	#NUM!	#NUM!	#NUM!	#NUM!	#NUM!		#NUM!	#NUM!	#NUM!	#NUM!	#NUM!	#NUM!									
MAPKAPK2	0.777	0.465	2.196	0.619	2.826	0.0034	X	4.125	0.137	4.004	0.149	0.971	0.2176									
CAMK2D	0.956	0.531	1.584	0.710	1.656	0.1522																
TSSK3	4.364	1.127	3.614	1.080	0.828	0.4521																
VRK1	#NUM!	#NUM!	#NUM!	#NUM!	#NUM!	#NUM!		#NUM!	#NUM!	#NUM!	#NUM!	#NUM!	#NUM!									
CAMK2A	1.383	0.227	1.691	0.340	1.223	0.1311																
CDKL4	#NUM!	#NUM!	#NUM!	#NUM!	#NUM!	#NUM!		#NUM!	#NUM!	#NUM!	#NUM!	#NUM!	#NUM!									
CDC2L6	0.720	0.658	1.049	0.498	1.457	0.3989																
DAPK2	1.965	0.317	1.081	0.736	0.550	0.0389	X	1.085	0.557	1.680	0.524	1.548	0.1203									
CAMKK2	1.514	0.249	1.142	0.120	0.754	0.0168	X	0.822	0.084	0.731	0.073	0.889	0.1053									
MAP4K1	1.042	0.285	1.428	0.158	1.371	0.0295	X	0.274	0.390	0.447	0.236	1.634	0.4197									
SNRK	6.978	1.852	6.991	1.699	1.002	0.9923																
ERBB4	0.841	0.167	0.751	0.156	0.893	0.4054																
CAMK4	1.515	1.422	1.239	0.371	0.818	0.6858																
BMX	1.244	0.062	1.237	0.260	0.995	0.9563																
FASTK	1.982	1.043	2.460	0.400	1.242	0.3661																
ALPK2	0.901	0.290	0.762	0.388	0.845	0.5383																
DYRK3	#NUM!	#NUM!	#NUM!	#NUM!	#NUM!	#NUM!		#NUM!	#NUM!	#NUM!	#NUM!	#NUM!	#NUM!									
ERN1	1.146	0.131	1.154	0.102	1.006	0.9232																
RPS6KC1	1.333	0.077	1.269	0.149	0.952	0.4169																
TAOK3	1.735	0.156	1.744	0.064	1.005	0.9036																
MAP3K13	1.700	0.312	1.183	0.268	0.696	0.0229	X	0.796	0.282	0.653	0.224	0.821	0.4015									
MAP4K5	#NUM!	#NUM!	#NUM!	#NUM!	#NUM!	#NUM!		2.966	0.886	2.514	1.064	0.848	0.4864									
MAST1	1.358	0.224	0.742	0.309	0.546	0.0069	X	0.709	0.176	0.838	0.197	1.183	0.3041									
BUB1B	2.294	0.911	2.644	0.279	1.152	0.4360																
CSNK2A2	#NUM!	#NUM!	#NUM!	#NUM!	#NUM!	#NUM!		#NUM!	#NUM!	#NUM!	#NUM!	#NUM!	#NUM!									
ADCK4	#NUM!	#NUM!	#NUM!	#NUM!	#NUM!	#NUM!		2.469	0.504	0.639	1.901	0.259	0.0712									
FLJ25006	#NUM!	#NUM!	#NUM!	#NUM!	#NUM!	#NUM!		#NUM!	#NUM!	#NUM!	#NUM!	#NUM!	#NUM!									
PRKD1	0.823	0.232	0.785	0.143	0.953	0.7589																
PDK1	#NUM!	#NUM!	#NUM!	#NUM!	#NUM!	#NUM!		1.730	0.696	1.280	0.782	0.740	0.3643									
PBK	#NUM!	#NUM!	#NUM!	#NUM!	#NUM!	#NUM!																
RIOK3	1.321	0.499	1.246	0.223	0.943	0.7673																
RPS6KB2	4.898	1.960	4.219	0.892	0.861	0.5007																
TTK	2.324	2.061	5.801	3.901	2.496	0.1820																
MAPK3	#NUM!	#NUM!	#NUM!	#NUM!	#NUM!	#NUM!																
NRBP1	1.411	0.626	0.907	0.677	0.643	0.2566																

ACVR1	3.033	1.173	0.855	2.691	0.282	0.1883																
DMPK	0.830	0.120	0.707	0.235	0.852	0.3279																
ADCK4	1.100	0.720	0.820	0.838	0.746	0.5870																
SRPK2	1.441	2.398	3.382	0.244	2.347	0.1572																
CSNK1G3	2.744	0.312	2.125	0.967	0.774	0.2100																
AKT3	#NUM!	#NUM!	#NUM!	#NUM!	#NUM!	#NUM!		0.928	0.502	1.132	0.455	1.221	0.5185									
STK16	0.887	0.535	0.209	0.937	0.235	0.1398																
VRK2	2.898	0.611	4.519	1.338	1.559	0.1292																
SRC	0.432	0.333	0.333	0.420	0.769	0.6882																
YES1	#NUM!	#NUM!	#NUM!	#NUM!	#NUM!	#NUM!		3.639	0.344	3.338	0.270	0.917	0.1620									
MKNK1	#NUM!	#NUM!	#NUM!	#NUM!	#NUM!	#NUM!		0.530	0.349	0.397	0.350	0.748	0.5625									
MAP2K3	0.768	0.851	0.624	0.811	0.813	0.8033																
MARK1	0.567	0.689	0.093	0.658	0.164	0.2980																
FLJ23356	#NUM!	#NUM!	#NUM!	#NUM!	#NUM!	#NUM!		1.467	0.442	1.815	0.531	1.238	0.2922									
PAK7	1.395	0.182	1.247	0.483	0.894	0.5398																
PRKAA1	2.855	0.984	0.795	1.079	0.278	0.0201	X	1.980	1.109	2.769	1.344	1.398	0.4006									
LYK5	#NUM!	#NUM!	#NUM!	#NUM!	#NUM!	#NUM!		1.110	2.277	2.057	0.698	1.852	0.4020									
PDK4	3.164	2.177	1.653	1.569	0.522	0.2638																
PNCK	3.568	2.679	#NUM!	#NUM!	#NUM!	#NUM!		#NUM!	#NUM!	#NUM!	#NUM!	#NUM!	#NUM!									
NEK6	#NUM!	#NUM!	#NUM!	#NUM!	#NUM!	#NUM!		#NUM!	#NUM!	#NUM!	#NUM!	#NUM!	#NUM!									
MET	4.374	3.223	5.042	2.710	1.153	0.7320																
MAPK14	2.160	1.780	1.602	1.048	0.742	0.6534																
NEK3	#NUM!	#NUM!	4.529	2.175	#NUM!	#NUM!		#NUM!	#NUM!	#NUM!	#NUM!	#NUM!	#NUM!									
PRKACA	1.274	1.829	1.408	1.522	1.106	0.9191																
NLK	0.992	0.530	1.454	0.627	1.466	0.0223	X	1.412	0.696	1.899	1.770	1.345	0.0476	X	X	0.461	2.171	0.859	1.711	1.864	0.7556	
RIOK2	#NUM!	#NUM!	#NUM!	#NUM!	#NUM!	#NUM!		2.136	1.048	3.572	1.294	1.672	0.1075									
RPS6KA6	4.669	0.206	3.720	0.191	0.797	0.0001	X	4.437	0.647	5.513	1.206	1.243	0.1167									
CHEK2	1.369	0.765	0.540	1.239	0.395	0.2987																
FASTKD2	2.395	1.551	1.644	0.668	0.686	0.4667																
CSK	2.224	2.336	0.932	1.078	0.419	0.3646																
BCKDK	#NUM!	#NUM!	#NUM!	#NUM!	#NUM!	#NUM!		1.611	0.290	1.490	0.422	0.925	0.6806									
CAMK1D	1.665	0.756	1.222	1.292	0.734	0.6349																
CSNK2A1	1.044	2.198	1.622	1.038	1.553	0.6156																
NEK7	#NUM!	#NUM!	#NUM!	#NUM!	#NUM!	#NUM!		#NUM!	#NUM!	#NUM!	#NUM!	#NUM!	#NUM!									
MAPK10	#NUM!	#NUM!	#NUM!	#NUM!	#NUM!	#NUM!		#NUM!	#NUM!	#NUM!	#NUM!	#NUM!	#NUM!									
PKMYT1	#NUM!	#NUM!	#NUM!	#NUM!	#NUM!	#NUM!		#NUM!	#NUM!	#NUM!	#NUM!	#NUM!	#NUM!									
PIM2	1.607	2.623	2.978	0.239	1.854	0.3381																
MAPKAPK3	0.289	3.550	1.935	0.811	6.705	0.4773																
MAPK13	2.233	1.414	4.639	2.225	2.077	0.1040																
RIOK1	1.667	0.544	1.506	0.282	0.904	0.5739																
CSNK1G1	#NUM!	#NUM!	#NUM!	#NUM!	#NUM!	#NUM!		#NUM!	#NUM!	#NUM!	#NUM!	#NUM!	#NUM!									
CDKL2	#NUM!	#NUM!	#NUM!	#NUM!	#NUM!	#NUM!		#NUM!	#NUM!	#NUM!	#NUM!	#NUM!	#NUM!									
MAPK6	#NUM!	#NUM!	#NUM!	#NUM!	#NUM!	#NUM!		#NUM!	#NUM!	#NUM!	#NUM!	#NUM!	#NUM!									
STK3	1.453	0.291	1.151	0.247	0.792	0.1157																
SYK	2.714	0.880	2.826	0.356	1.041	0.7985																
PCTK1	3.452	0.542	0.981	2.605	0.284	0.0738																
BMP2K	#NUM!	#NUM!	#NUM!	#NUM!	#NUM!	#NUM!		#NUM!	#NUM!	#NUM!	#NUM!	#NUM!	#NUM!									
RP6-213H19	#NUM!	#NUM!	#NUM!	#NUM!	#NUM!	#NUM!		#NUM!	#NUM!	#NUM!	#NUM!	#NUM!	#NUM!									
OXSR1	#NUM!	#NUM!	#NUM!	#NUM!	#NUM!	#NUM!		#NUM!	#NUM!	#NUM!	#NUM!	#NUM!	#NUM!									
SNF1LK	#NUM!	#NUM!	#NUM!	#NUM!	#NUM!	#NUM!		#NUM!	#NUM!	#NUM!	#NUM!	#NUM!	#NUM!									
CSNK1G1	1.485	0.444	1.543	0.766	1.039	0.8865																
CSNK1G2	#NUM!	#NUM!	#NUM!	#NUM!	#NUM!	#NUM!		#NUM!	#NUM!	#NUM!	#NUM!	#NUM!	#NUM!									
FASTKD5	#NUM!	#NUM!	#NUM!	#NUM!	#NUM!	#NUM!		#NUM!	#NUM!	#NUM!	#NUM!	#NUM!	#NUM!									
SCYL3	0.600	1.140	0.910	0.696	1.516	0.6288																
PCTK1	6.869	2.993	3.896	2.116	0.567	0.1807																
MARK3	4.830	1.416	4.033	1.320	0.835	0.5150																
SYK	1.888	0.260	1.360	0.362	0.721	0.0295	X	0.746	0.651	2.032	0.828	2.722	0.0260	X								
RPS6KB1	2.348	0.796	2.858	1.136	1.217	0.4347																
CDK5	3.725	2.035	4.266	1.692	1.145	0.6973																
PDK2	#NUM!	#NUM!	#NUM!	#NUM!	#NUM!	#NUM!		#NUM!	#NUM!	#NUM!	#NUM!	#NUM!	#NUM!									
MAPK9	2.604	1.009	0.096	3.105	0.037	0.1691																
TRIB1	0.023	2.415	1.957	0.579	84.707	0.1198																
CDK2	0.869	0.972	0.571	0.186	0.657	0.0470	X	0.286	0.788	1.079	1.198	3.775	0.2515									
PRKG1	0.675	1.311	1.494	0.205	2.214	0.2049																
PLK1	1.490	1.816	3.969	0.743	2.664	0.0528																
MAPKAPK5	0.914	0.955	1.314	0.791	1.438	0.4908																
STK25	0.740	2.333	3.077	0.576	4.157	0.0644																
STK40	0.757	0.764	1.032	1.041	1.362	0.6475																
PRPF4B	0.095	0.620	0.556	0.576	5.887	0.2568																
MKNK2	1.711	0.736	1.388	0.541	0.811	0.4513																
MAPK12	1.322	0.250	0.846	0.202	0.640	0.0107	X	1.727	1.097	0.504	0.707	0.292	0.0987			0.918	0.169	0.816	0.224	0.890	0.4426	
AAK1	4.100	0.278	4.215	0.564	1.028	0.6945																
STK32B	1.039	0.174	1.117	0.413	1.075	0.7076																
TLK1	4.296	0.761	3.707	0.327	0.863	0.1507																
STK19	0.235	0.122	0.323	0.153	1.371	0.3470																
SCYL2	1.367	0.490	0.830	0.468	0.607	0.1146																
ZAP70	#NUM!	#NUM!	#NUM!	#NUM!	#NUM!	#NUM!		#NUM!	#NUM!	#NUM!	#NUM!	#NUM!	#NUM!									
CASK	4.782	0.140	4.139	0.930	0.866	0.1648																
RPS6KA3	0.642	0.416	0.836	0.440	1.301	0.4963																
YSK4	3.234	2.887	5.371	0.871	1.661	0.2870																
CAMK1	#NUM!	#NUM!	#NUM!	#NUM!	#NUM!	#NUM!		3.055	2.593	2.634	2.953	0.862	0.8374									
SGK493	2.289	2.029	2.403	2.353	1.050	0.9524																
CDC2	#NUM!	#NUM!	#NUM!	#NUM!	#NUM!	#NUM!		#NUM!	#NUM!	#NUM!	#NUM!	#NUM!	#NUM!									
SIK2	3.432	0.556	2.755	1.621	0.803	0.4557																
MAPK1	0.741	1.800	0.071	0.810	0.096	0.0719																
PAK1	#NUM!	#NUM!	#NUM!	#NUM!	#NUM!	#NUM!		#NUM!	#NUM!	#NUM!	#NUM!	#NUM!	#NUM!									
STK24	1.369	0.638	1.283	0.919	0.937	0.8678																
TRIB2	#NUM!	#NUM!	#NUM!	#NUM!	#NUM!	#NUM!		#NUM!	#NUM!	#NUM!	#NUM!	#NUM!	#NUM!									
NRBP	1.723	0.430	0.773	1.496	0.449	0.2095																
STK40	1.013	0.793	1.023	1.126	1.010	0.9877																
ADCK5	5.779	0.482	7.260	1.178	1.256	0.0315	X	4.043	1.094	2.307	0.859	0.571	0.0468	X								
EEF2K	#NUM!	#NUM!	#NUM!	#NUM!	#NUM!	#NUM!		#NUM!	#NUM!	#NUM!	#NUM!	#NUM!	#NUM!									
PDK3	#NUM!	#NUM!	#NUM!	#NUM!	#NUM!	#NUM!		#NUM!	#NUM!	#NUM!	#NUM!	#NUM!	#NUM!									
PXK	2.922	0.939	1.219	1.801	0.417	0.1331																
SGK196	1.629	0.645	0.843	0.524	0.517	0.0673																
EPHA7	1.219	0.711	0.431	1.037	0.353	0.2169																
TLK2	#NUM!	#NUM!	#NUM!	#NUM!	#NUM!	#NUM!		3.306	1.172	0.543	0.344	0.164	0.0083	X								
STRADA	1.035	0.842	0.945	0.693	0.913	0.8680																
MAPK8	#NUM!	#NUM!	#NUM!	#NUM!	#NUM!	#NUM!		#NUM!	#NUM!	#NUM!	#NUM!	#NUM!	#NUM!									
STK19	1.201	2.413	2.597	0.574	2.162	0.3848																
CDC2L2	#NUM!	#NUM!	#NUM!	#NUM!	#NUM!	#NUM!		3.073	1.361	3.442	0.520	1.120	0.6801									
BRSK2	3.631	1.303	1.145	1.058	0.315	0.0434	X	1.135	1.259	3.083	0.968	2.716	0.0337	X								
MARK2	#NUM!	#NUM!	#NUM!	#NUM!	#NUM!	#NUM!		#NUM!	#NUM!	#NUM!	#NUM!	#NUM!	#NUM!									
PAK4	#NUM!	#NUM!	#NUM!	#NUM!	#NUM!	#NUM!		#NUM!	#NUM!	#NUM!	#NUM!	#NUM!	#NUM!									
MAP2K2	#NUM!	#NUM!	#NUM!	#NUM!	#NUM!	#NUM!		1.330	0.724	1.128	0.557	0.849	0.6351									
**420**							**81**							**34**	**17**							**3**
**total**							**total**							**total**	**total**							**total**

*Exp1 results were repeated in Exp 2 IF t-test <0.05 in Exp 1; also IF Exp 1 yielded "#NUM!" (denoting values at or below background, then it was repeated in Exp 2.

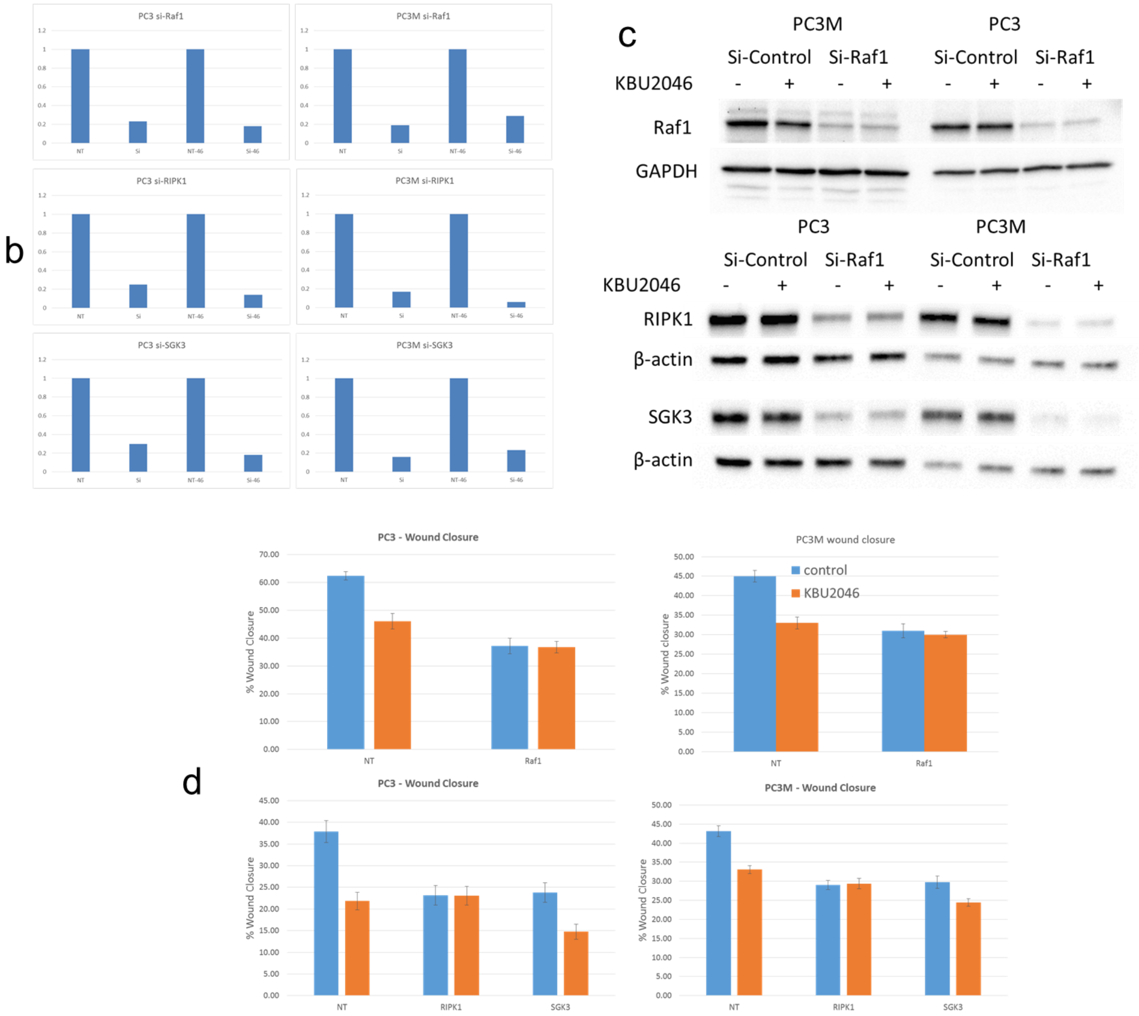

Supplementary Figures 17. KBU2046-mediated changes in the signature of client proteins bound to HSP90β/CDC37 mediate effects upon cell motility.

LUMIER assay (**a**). HEK293T cells stably transfected with HSP90β-luciferase were transfected with 1 of 420 different FLAG-tagged protein kinases, treated with 10 µM KBU2046 for 3 days, and LUMIER assays performed as described [12]. All experiments had N = 5 vehicle treated control plus N = 5 KBU2046 treated separate wells of cells. Those yielding a t-test p value <0.05

in experiment #1, or those whose values were at or below baseline, were repeated in experiment #2. Those yielding a t-test p value <0.05 in both experiments and exhibiting a change in hetercomplex association with KBU2046 treatment in the same direction in both experiments were selected. Of 420 kinase proteins tested, 17 (4%) met these criteria (graphically depicted in manuscript Fig. 6a). For these 17 kinases, the experiment was repeated except that HEK293T cells were treated with TGFβ for the last 24 hrs of the 3 day incubation (graphically depicted in manuscript Fig. 6b). Wound healing assay (**b-d**). PC3 or PC3M cells were treated +/- KBU2046 and with siRNA to the denoted gene, or with non-targeting control siRNA (NT), and effects upon transcript (**b**) or protein expression (**c**) measured by qRT/PCR or Western blot, respectively. For qRT/PCR, values are expressed as the target gene/GAPDH ratio, normalized to that of NT/-KBU2046 cells. Under the same conditions, wound healing assays were performed (**d**).

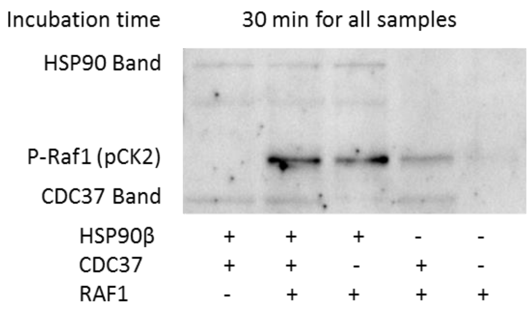

Supplementary Figure 18. KBU2046 affects client protein function and interaction with HSP90β/CDC37 heterocomplexes *in vitro*.

RAF1, CDC37 and HSP90β proteins were added, as denoted, in a RAF1 *in vitro* kinase assay, and autophosphorylation of RAF1 detected by Western blot with phospho-CK2 antibody. Non-

specific binding to HSP90β and CDC37 proteins provides a measure of their presence and equal amounts for loading controls.

Whole blot for manuscript Fig. 6 c

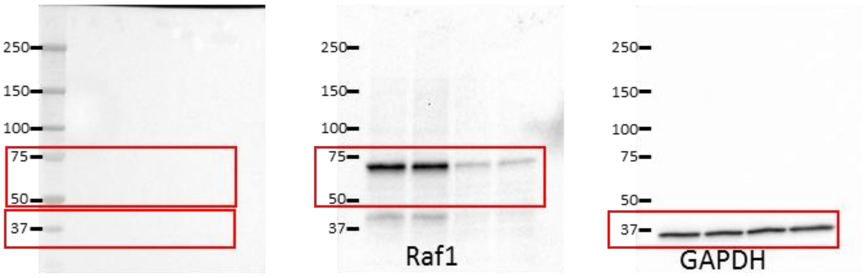

Whole blot for manuscript Fig. 6 e

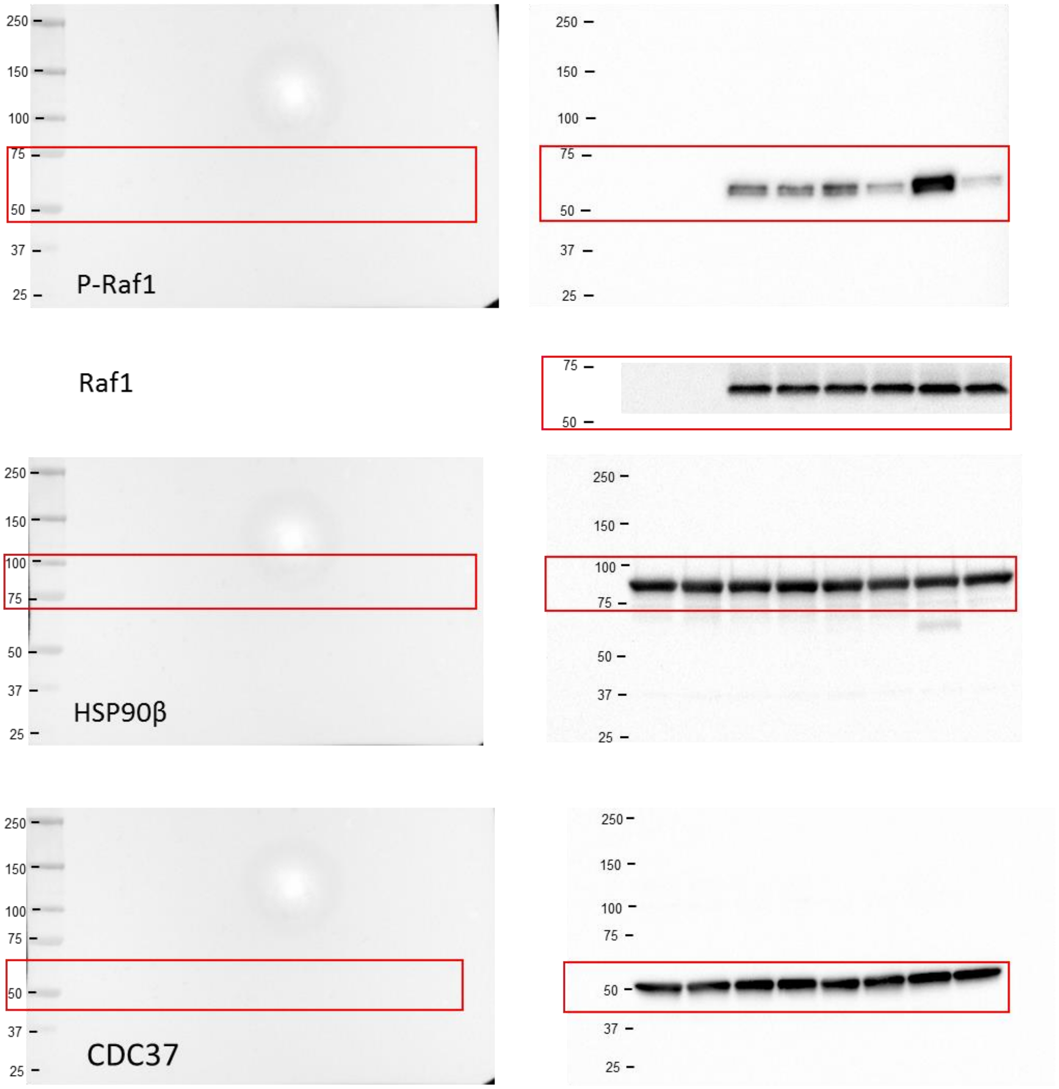

Whole blots for manuscript Fig. 6f

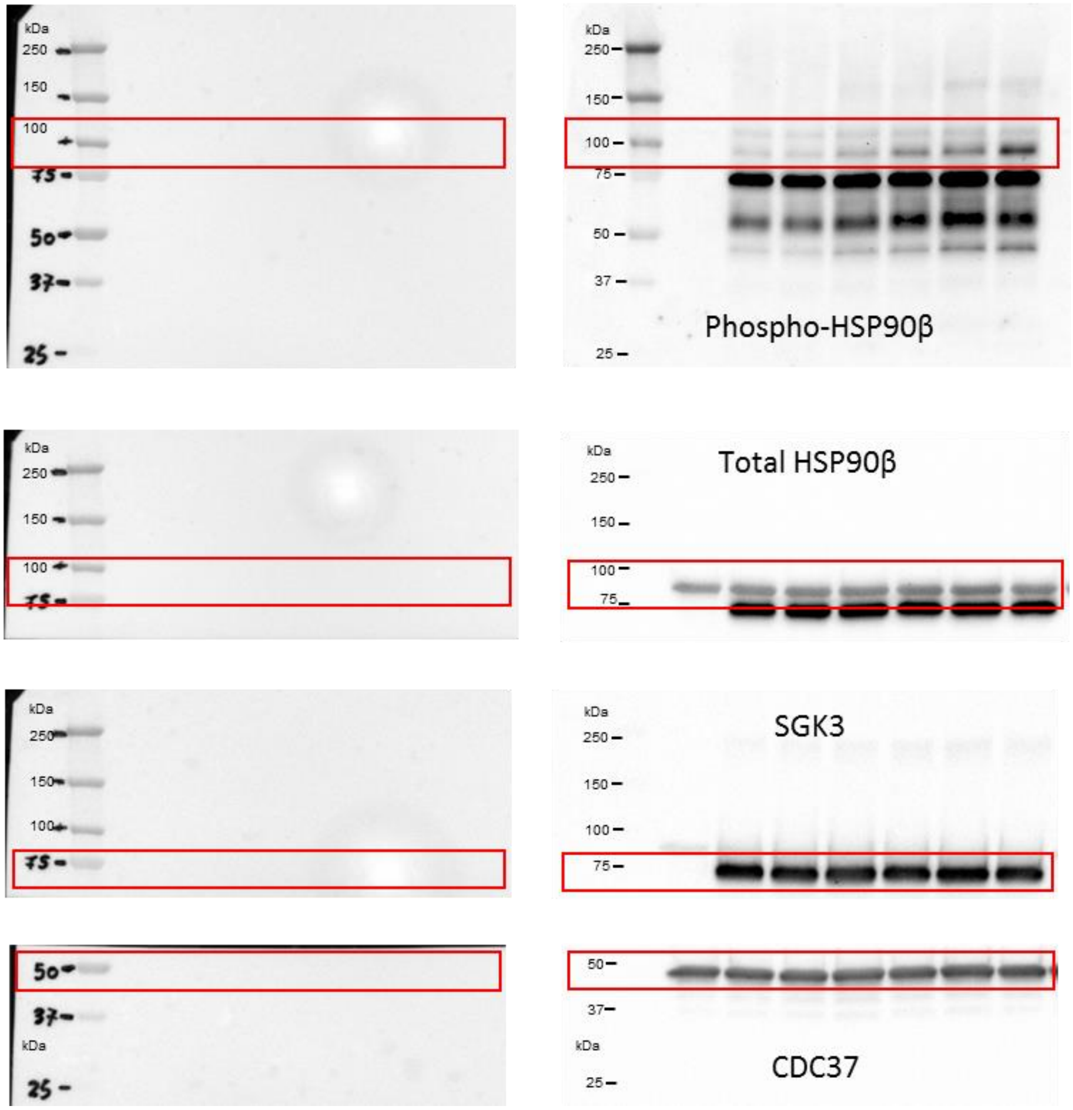

Whole blots for manuscript Fig 6g

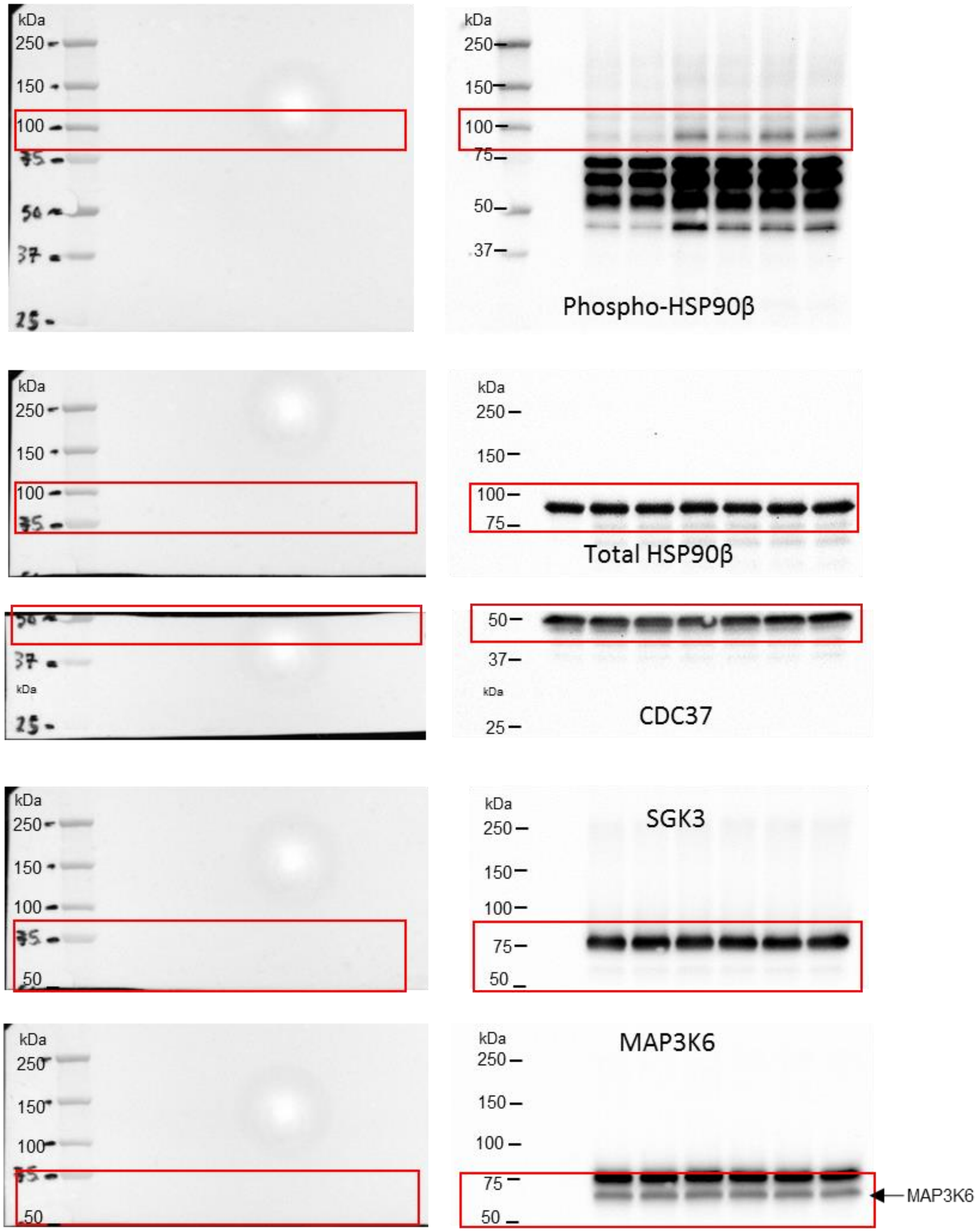

Supplementary Figure 19. Uncropped scans of the most important blots.

Uncropped scans for manuscript figs 6c, e, f and g are depicted. All images were acquired using a ChemiDoc XRS imaging system (Bio-Rad, Hercules, CA). Images for protein standards (for molecular weight) were obtained using the white light image capture function and the corresponding images of the HRP-secondary antibody-primary antibody complexes directed at associated proteins of interest were visualized with the chemiluminescence image capture function. Molecular weight markers from the white light images were utilized to assign size references to the corresponding chemiluminescence images. Bands of interest are outlined in red on the whole blot images.

Supplementary Notes.

Supplementary Note 1. The synthetic strategy. The schematic flow of our synthetic strategy is depicted in manuscript Figure 1a. Beginning with 4´,5,7-trihydroxyisoflavone (genistein) as a chemical scaffold, we pursued a fragment-based chemical diversification synthesis approach, and coupled this in an iterative fashion to biological assays of cell invasion and cell growth inhibition. Compounds that inhibited cell invasion but did not inhibit cell growth were selected for further modification and evaluation.

The initial round of synthesis was designed to examine the removal of individual chemical fragments. In this manner, the importance of these functional groups in mediating efficacy (inhibition of cell invasion) could be determined. Subsequent rounds built upon refined structure activity relationship (SAR) knowledge, and sought to improve efficacy, while deselecting for

toxicity (cell growth inhibition). Initial assays were performed with PC3 and PC3-M cells (PC3 cell data not shown). However, as these studies yielded similar findings, subsequent screening assays utilized only PC3-M cells. In designing chemical synthetic routes, priority was given to efficacy and toxicity parameters. Additional design features were also included in our chemical synthetic routes, but they were only incorporated if they did not compromise efficacy and toxicity parameters. These additional design features included removal of fragments that mediated genistein binding to the estrogen receptor (ER), as determined by ER-genistein 3D x-ray crystal structures (Protein Data Base IDs:1X7R and 1X7J, for crystal structures of ERα and ERβ with bound genistein, respectively). These features also included removal of chemical fragments that are considered to increase susceptibility to rapid metabolism, especially that by the cytochrome P450 (CYP) pathway. The final feature involved incorporation of chemical characteristics previously shown to be associated with effective drugs and which together generally impart more favorable pharmacologic properties, including those described by Lipinski et al. [2].

Supplementary Note 2. KBU2046 does not inhibit kinase or phosphatase activity. Three different assays systems were employed.

Kinase assay system #1. The KINOMEscan™ assay (Ambit Biosciences). This assay evaluates 442 kinases, including 400 distinct parental kinases plus mutants known to alter activity or responsiveness. It does so in the context of an assay that measures the ability of putative inhibitors to inhibit binding of bacterial purified kinase to immobilized phospho-acceptor protein substrate. This approach has been successfully used by multiple groups to identify kinase interactions of several small molecule kinase inhibitors [13, 14]. KBU2046 was tested at 10 μM.

This assay was completely negative (data not shown). There were two initial false positives (for a false positive rate of 0.4%), that were subsequently found to be negative in a dedicated follow up analysis. Specific and important negative findings include MKK4, p38 MAPK (α, β, γ and δ isoforms) and RAF1. Conclusions: there was no evidence that KBU2046 inhibits kinase function by competing for phospho-acceptor binding.

Kinase assay system #2. The Kinex™ kinase assay system (Kinexus Proteomics Company). This platform uses recombinant human protein kinases expressed in an insect expression system, thus allowing an avenue for post-translational modification. Also, this platform measures inhibition of ATP binding. This platform putatively measured 200 different kinases, and we tested KBU2046 at 1 and 10 μM. This assay was also completely negative. There were two important technical issues. First, at 10 μM KBU2046 interfered with the colorimetric-based readout of the assay. Second, we found that 1/3rd of the control enzymes were not active. However, while considering the above factors, this screen was informative for a number of kinases (where controls were active and at KBU2046 concentrations that did not interfere with detection). In this regard, we want to highlight that KBU2046 did not inhibit p38 MAPK (all isoforms), MKK4 or RAF1. Conclusions: there was no evidence that KBU2046 inhibits kinase function by competing for ATP binding in the active site.

Kinase assay system #3. KinaseProfiler™ and PhosphataseProfiler™ assay platforms (Millipore). This platform is radiometric-based (considered gold standard). It measures competition with respect to ATP for kinases and substrate protein for phosphatases. Most proteins were expressed via an insect-based system, and it evaluates a panel of 284 kinases and 20 phosphatases. There were two initial false positives, for a false positive rate of 0.7%, but both failed to be confirmed upon in-depth investigation. Important negative findings include: MKK4,

MKK6, p38 MAPK, MAPKAPK2, RAF1, ERK, MEK1, JNK1, 2 and 3, and numerous other MAPK cascade-associated kinases. Conclusions: there is no evidence that KBU2046 inhibits kinase activity by competing for ATP binding in the active site. No evidence supports inhibition of phosphatase activity.

Supplementary Methods.

Construction of structural model of HSP90β, CDC37 and KBU2046 interaction.

Our analysis began with experimentally determined structural information, including the crystal structures of human HSP90β (PDBs 1uym, 3nmq and 3pry) and HSP82-CDC37 complex from yeast (PDB 1us7), which were determined by X-ray diffraction-based crystallographic analysis. We experimentally probed the HSP90β structure using chemical cross-linking with mass spectrometry, employing chemical cross-linkers of various lengths, as previously described by us [6]. Cross-linked peptide samples were analyzed using ReACT [15] which allows targeted MS^3 to be carried out efficiently on each released peptide that satisfies expected PIR mass relationships [16]. Further, the chemical structure of KBU2046 had been experimentally determined, as described above. Finally, this experimental information was integrated using the APPLIED Pipeline (Analysis Pipeline for Protein Ligand Interactions and Experimental Determination) at the Argonne Leadership Computing Facility, Argonne National Laboratory, tuned for the 786,432 core BlueGene/Q Mira [17], using a multi-stage pipeline that considers protein-protein/ligand interactions through evolutionary protein surface analysis [18, 19, 20], robust homology modeling [21], massively parallel docking simulations using mixed strategies [22, 23, 24, 25, 26], and advanced, physics-based rescoring methodologies [26, 27, 28], all as described by us.

We had found that KBU2046 does not bind directly to HSP90β or CDC37 (Supplementary Fig. 14), but that it does bind to the HSP90β-CDC37 complex (manuscript Fig. 5a). Therefore, the complex must afford a suitable binding pocket that is not independently present on either protein. We began with homology based modeling using existing structures from the protein data bank (PDB). Structures of the human HSP90β N-terminal ATPase domain (PDB ids=1uym, 3nmq) and middle domain (PDB id=3pry) were used. The noncontiguous models cover 65% of the primary sequence, separated by a highly disordered region of 63 residues that terminates the ATP binding domain. No experimental models exist for the C-terminal region. The entirety of the HSP90β structure was then modeled against the HSP82 template from *S. cerevisiae* (PDB id=2cg9) [21]. A model of the complex of HSP90β-CDC37 was completed through superposition of the HSP90β model onto the structure of the *S. cerevisiae* HSP82-CDC37 complex (PDB id=1us7). The sequence identity between HSP90β and HSP82 is 94% at the CDC37 interface (86% for entire protein), thus preserving the integrity of the interactions.

Supplementary References

1. Shoemaker RH. The NCI60 human tumour cell line anticancer drug screen. *Nature reviews Cancer* **6**, 813-823 (2006).

2. Lipinski CA, Lombardo F, Dominy BW, Feeney PJ. Experimental and computational approaches to estimate solubility and permeability in drug discovery and development settings. *Adv Drug Deliv Rev* **46**, 3-26 (2001).

3. Kataria BK, *et al.* The pharmacokinetics of propofol in children using three different data analysis approaches. *Anesthesiology* **80**, 104-122 (1994).

4. Knodell RG, *et al.* Formulation and application of a numerical scoring system for assessing histological activity in asymptomatic chronic active hepatitis. *Hepatology* **1**, 431-435 (1981).

5. Krishna SN, *et al.* A fluorescence-based thermal shift assay identifies inhibitors of mitogen activated protein kinase kinase 4. *PloS one* **8**, e81504 (2013).

6. Chavez JD, Weisbrod CR, Zheng C, Eng JK, Bruce JE. Protein interactions, post-translational modifications and topologies in human cells. *Molecular & cellular proteomics : MCP* **12**, 1451-1467 (2013).

7. Makowska-Grzyska M, *et al.* Bacillus anthracis inosine 5'-monophosphate dehydrogenase in action: the first bacterial series of structures of phosphate ion-, substrate-, and product-bound complexes. *Biochemistry* **51**, 6148-6163 (2012).

8. Wright L, *et al.* Structure-activity relationships in purine-based inhibitor binding to HSP90 isoforms. *Chemistry & biology* **11**, 775-785 (2004).

9. Binkowski TA, Naghibzadeh S, Liang J. CASTp: Computed Atlas of Surface Topography of proteins. *Nucleic acids research* **31**, 3352-3355 (2003).

10. Roe SM, *et al.* The Mechanism of Hsp90 regulation by the protein kinase-specific cochaperone p50(cdc37). *Cell* **116**, 87-98 (2004).

11. Vaughan CK, *et al.* Structure of an Hsp90-Cdc37-Cdk4 complex. *Molecular cell* **23**, 697-707 (2006).

12. Taipale M, *et al.* Quantitative analysis of HSP90-client interactions reveals principles of substrate recognition. *Cell* **150**, 987-1001 (2012).

13. Fabian MA, *et al.* A small molecule-kinase interaction map for clinical kinase inhibitors. *Nat Biotechnol* **23**, 329-336 (2005).

14. Karaman MW, *et al.* A quantitative analysis of kinase inhibitor selectivity. *Nat Biotechnol* **26**, 127-132 (2008).

15. Weisbrod CR, Chavez JD, Eng JK, Yang L, Zheng C, Bruce JE. In Vivo Protein Interaction Network Identified with a Novel Real-Time Cross-Linked Peptide Identification Strategy. *Journal of proteome research*, (2013).

16. Tang X, Munske GR, Siems WF, Bruce JE. Mass spectrometry identifiable cross-linking strategy for studying protein-protein interactions. *Analytical chemistry* **77**, 311-318 (2005).

17. Zhao Y, Wilde M, Foster I. Virtual Data Language: A Typed Workflow Notation for Diversely Structured Scientific Data. In: *Workflows for eScience: Scientific Workflows for Grids* (ed^(eds Editors:, Taylor IJ, Deelman E, Gannon DB, Shields M). Springer (2007).

18. Binkowski TA, Adamian L, Liang J. Inferring functional relationships of proteins from local sequence and spatial surface patterns. *Journal of molecular biology* **332**, 505-526 (2003).

19. Binkowski TA, Joachimiak A. Protein functional surfaces: global shape matching and local spatial alignments of ligand binding sites. *BMC structural biology* **8**, 45 (2008).

20. Binkowski TA, Joachimiak A, Liang J. Protein surface analysis for function annotation in high-throughput structural genomics pipeline. *Protein science : a publication of the Protein Society* **14**, 2972-2981 (2005).

21. Leaver-Fay A*, et al.* ROSETTA3: an object-oriented software suite for the simulation and design of macromolecules. *Methods in enzymology* **487**, 545-574 (2011).

22. Lang PT*, et al.* DOCK 6: combining techniques to model RNA-small molecule complexes. *RNA* **15**, 1219-1230 (2009).

23. Graves AP, Shivakumar DM, Boyce SE, Jacobson MP, Case DA, Shoichet BK. Rescoring docking hit lists for model cavity sites: predictions and experimental testing. *Journal of molecular biology* **377**, 914-934 (2008).

24. Morris GM, *et al.* AutoDock4 and AutoDockTools4: Automated docking with selective receptor flexibility. *Journal of computational chemistry* **30**, 2785-2791 (2009).

25. Deng Y, Roux B. Computation of binding free energy with molecular dynamics and grand canonical Monte Carlo simulations. *The Journal of chemical physics* **128**, 115103 (2008).

26. Wang J, Deng Y, Roux B. Absolute binding free energy calculations using molecular dynamics simulations with restraining potentials. *Biophysical journal* **91**, 2798-2814 (2006).

27. Jiang W, Hodoscek M, Roux B. Computation of Absolute Hydration and Binding Free Energy with Free Energy Perturbation Distributed Replica-Exchange Molecular Dynamics (FEP/REMD). *Journal of chemical theory and computation* **5**, 2583-2588 (2009).

28. Jiang W, Roux B. Free Energy Perturbation Hamiltonian Replica-Exchange Molecular Dynamics (FEP/H-REMD) for Absolute Ligand Binding Free Energy Calculations. *Journal of chemical theory and computation* **6**, 2559-2565 (2010).
